# Patient‐reported outcome measures in pediatrics: An overview of reviews

**DOI:** 10.1002/pdi3.77

**Published:** 2024-06-01

**Authors:** Ruobing Lei, Jin Xiong, Haiyun Wang, Yuehuan Li, Janne Estill, Qiu Li, Yaolong Chen

**Affiliations:** ^1^ Chevidence Lab of Child & Adolescent Health Children’s Hospital of Chongqing Medical University Chongqing China; ^2^ National Clinical Research Center for Child Health and Disorders Chongqing China; ^3^ Ministry of Education Key Laboratory of Child Development and Disorders Chongqing China; ^4^ China International Science and Technology Cooperation Base of Child Development and Critical Disorders Chongqing China; ^5^ Chongqing Key Laboratory of Pediatrics Chongqing China; ^6^ College of Pediatrics Chongqing Medical University Chongqing China; ^7^ Department of Cardiac Surgery Beijing Anzhen Hospital Capital Medical University Beijing China; ^8^ Institute of Global Health University of Geneva Geneva Switzerland; ^9^ Department of Nephrology Children's Hospital of Chongqing Medical University Chongqing China; ^10^ Research Unit of Evidence‐Based Evaluation and Guidelines Chinese Academy of Medical Sciences (2021RU017) School of Basic Medical Sciences Lanzhou University Lanzhou China; ^11^ WHO Collaborating Center for Guideline Implementation and Knowledge Translation Lanzhou China

**Keywords:** overviews, patient‐reported outcome measures, pediatrics

## Abstract

Patient‐reported outcome measures (PROMs) are standardized and validated self‐administered questionnaires for assessing patients' overall well‐being, disease burden, and health‐related quality of life. For children, their cognitive development, reading ability and language skills need to be considered when selecting the optimal PROM. High‐quality systematic reviews (SRs) can provide a comprehensive overview of the available PROMs and provide evidence‐based recommendations for pediatricians. Therefore, this study aims to provide an overview of pediatric SRs of PROMs. PubMed, Embase and Cochrane Library were searched to identify SRs of PROMs published in English focusing on the health of children and adolescents. Four researchers performed literature screening and data extraction, and evaluated the methodological quality of SRs using the A MeaSurement Tool to Assess systematic Reviews tool. Forty‐four SRs of PROMs published between 2006 and 2022 were included, recommending 123 PROMs, of which the most recommended were the pediatric quality of life inventory and its subscales and the EuroQol five dimension questionnaire. Thirty‐six conditions were addressed; the most frequent ICD‐11 category was “Mental, behavioral or neurodevelopmental disorders” (*n* = 9, 20.5%). The PROMs covered nine categories of contents to measure, the most frequent being the quality of life (*n* = 37, 30.1%). Content validity (*n* = 67, 54.5%) and internal consistency (*n* = 65, 52.9%) were the most commonly reported and measurement error (*n* = 10, 8.1%) was the least. The methodological and reporting of psychometric properties for SRs need further improvement. In addition, reporting of details such as the age when children should self‐report the measures needs also improvement.

## Graphical abstract

This study found that the diversity in the target diseases and measurement content was limited in pediatric PROMs. The age from which children should self‐report the measurements differed across PROMs, and some definitions were unclear. In addition, reporting of psychometric properties for SR needs improvement, and tailored tools for evaluating the methodological quality of SRs on PROMs are needed.

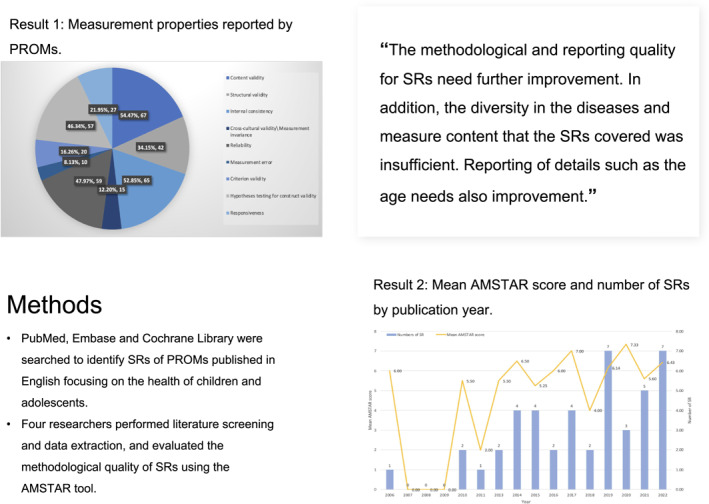

## 1 INTRODUCTION

The U.S. Food and Drug Administration classifies clinical outcomes into four categories: patient‐reported outcomes (PROs), observer‐reported outcomes, clinician‐reported outcomes, and Performance outcomes.^1^ PROs are reported directly by the patient, without interpretation by a clinician or anyone else, and relate to the patient's health, quality of life, or healthcare‐ or treatment‐related functional status. With the development of the “patient‐centered” model of care,^2^ PROs are considered to help clinicians better understand their patients' health status and treatment goals.^3^, ^4^ Patient‐reported outcome measures (PROMs) are standardized and validated self‐reported measurements used to quantify PROs in the areas of physical, emotional, social, functional, and overall well‐being, disease burden, and health‐related quality of life (HRQOL).^5^ Integrating PROMs into routine clinical practice can enhance communication between healthcare providers, patients and caregivers, leading to better long‐term care management and patient outcomes.^6^

The health goals, health risks faced, and clinical context of health care are very different in pediatrics compared with adults,^7^ which makes the design of PROMs for pediatric populations is more complex and comes in many different forms.^8^ There are also many challenges in applying pediatric PROMs, such as the need to consider the vocabulary, cognitive and reading abilities of children of different ages. The initial view that children's self‐reported information is unreliable^8^ has been followed by studies showing that children over 8 years of age can reliably report their health status^9^, ^10^; Meanwhile, young children with other special conditions need proxy reports from parents or caregivers,^11^ but it is also argued that proxy reports do not accurately reflect children's symptoms and experiences, especially psychological symptoms.^12^ Therefore, it is important to select the PROMs that best reflect the health status of children according to their age, diseases, and cognitive abilities.

The methodological quality of currently available pediatric PROMs varies widely. One study used the COnsensus‐based Standards for the selection of health Measurement INstruments (COSMIN) tool to evaluate measurement instruments in the area of self‐care for healthy children and found that the evidence available does not allow firm conclusions about the instruments identified in terms of reliability and validity.^13^ Of the 25 generic, multidimensional PROMs developed for children and young people, only 12 PROMs were of sufficiently high quality to be recommended.^14^

Systematic reviews (SRs) are an important way to select the most appropriate PROMs for different situations.^15^ SRs of PROMs include and analyze studies which validated measurement properties of one or more PROMs, such as content validity, structural validity and internal consistency. High‐quality SRs can provide a comprehensive overview of the measurement properties of PROMs and provide pediatricians with evidence‐based recommendations for the selection of the most appropriate PROMs. Therefore, this study aims to retrieve SRs of pediatric PROMs, evaluate the methodological quality of the SRs, and integrate and analyze the characteristics of the SRs and measurement properties of the recommended pediatric PROMs.

## 2 METHODS

We followed the guidance for overviews of reviews published by Hunt et al.^16^ We also reported this overview according to the PRIOR statement.^17^

### 2.1 Eligibility criteria

We included studies that met the following inclusion criteria: (1) the study focused on the health of children and adolescents under the age of 18^18^; and (2) the study was a SRs of pediatric PROMs.

The following types of studies were excluded: (1) studies that included both children and adults if the findings on PROMs for pediatrics could not be extracted, or if the applicable population was not explicitly described; (2) studies that did not evaluate the validity and reliability of PROMs; and (3) studies that did not make explicit recommendations based on the evidence.

### 2.2 Information sources and search strategy

On February 14, 2023, two reviewers independently searched three academic databases (MEDLINE, Embase, Cochrane Library) using search terms such as “patient reported outcome measures,” “quality of life,” “instruments,” “children,” “systematic review.” The details of the search strategies are shown in Table S1. In addition, we searched Google and the website of the COSMIN initiative.

### 2.3 Selection process

The records were divided for two pairs of investigators (RL & YL, HW & JX). Both investigators independently screened the titles and abstracts using the bibliographic software EndNote, and then screened the full texts of potentially eligible articles to determine the inclusion. Disagreements were resolved by consensus and discussion with a third reviewer (YC). A pilot test was performed prior to the screening to reach agreement on the screening process.

### 2.4 Data collection process

The following data were extracted by two reviewers (HW & JX) independently using a standardized form from each included SR: (1) characteristics of included SR: publication year; condition; ICD‐11 (International Classification of Diseases 11th Revision)^19^ (Reviewers judge or consult experts for advice according to the content of SRs); number of included study; number of included PROMs; name of criteria; (2) characteristics of the recommended PROMs: name and acronym; year developed; country of development; available language versions; time to complete (min); content of interest (quality of life, body functions, emotional functions, exercise tolerance functions, executive functions, sleep functions, social functions, behavior, sensory functions; based on the model of Wilson & Cleary^20^ and International classification of functioning, disability and health: children and youth version [ICF‐CY]^21^); population; mode of reporting; number of items; domains assessed; (3) measurement properties. Disagreements were resolved by consensus and discussion with a third reviewer (YC).

### 2.5 Quality appraisal

Two reviewer teams separately and independently evaluated the methodological quality of the included SRs, using “A MeaSurement Tool to Assess systematic Reviews” (AMSTAR) instrument^22^ is now a recognized tool for assessing the methodological quality of SRs. In SRs of PROMs, publication bias is not applicable in studies on measurement properties, so the maximum AMSTAR score is 10 points, with studies scoring between 8 and 10 being considered as high quality, studies scoring between 5 and 7 as medium quality, and studies scoring between 0 and 4 as low quality.^23^ Disagreements were resolved by consensus and discussion with a third reviewer (YC).

### 2.6 Data analysis

The characteristics of the included SRs were recorded into an Excel spreadsheet (Microsoft Corporation). Frequencies and percentages were used to describe characteristics and AMSTAR scores.^24^

## 3 RESULTS

**FIGURE 1:**
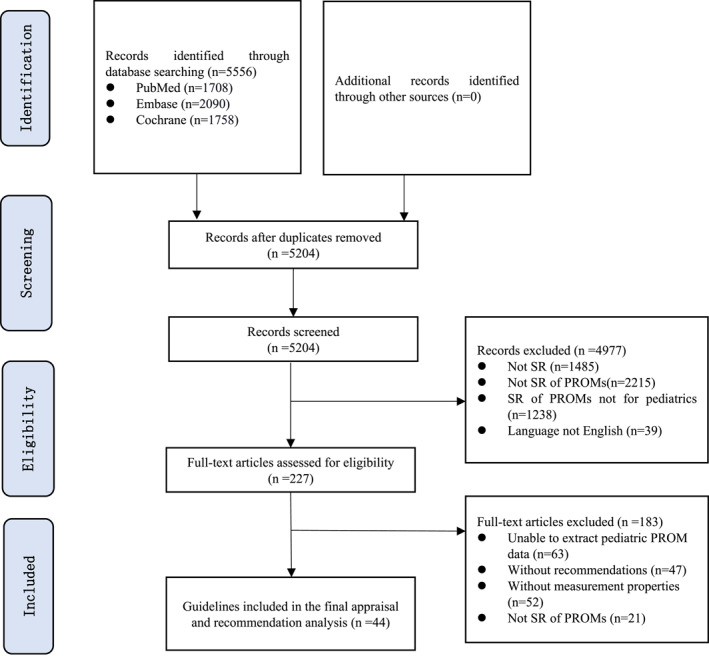
Flow diagram for inclusion and exclusion of studies.

A total of 5556 records were retrieved, including 1708 from PubMed, 2090 from Embase, and 1758 from Cochrane Database. After removing 352 duplicates, 4977 records were excluded based on the screening of titles and abstracts. The full texts of 227 records were screened, of which 183 were excluded (Table S2) and 44 included. The selection process is shown in Figure 1.

### 3.1 Characteristics of the included SRs

A total of 1888 studies containing 635 different PROMs were included in the 44 SRs, in which 123 PROMs were recommended. Publication years of SRs ranged between 2006 and 2022, with seven (15.9%) SRs published in both 2019 and 2022, followed by 5 (11.4%) published in 2021. The highest number of recommended PROMs was in 2021 with 25 (20.3%) recommended PROMs, followed by 19 (15.5%) in 2022 and 17 in 2019 (Table 1).

**TABLE 1:** Characteristics of included studies.

Study ID	Publication year	Target condition	Category of target category (ICD‐11)	Number of included studies	Number of included PROMs	Number of recommended PROMs	Name of criteria to evaluate the measurement properties
Kusi‐Mensah 2022	2022	Brain pathology	Diseases of the nervous system	51	49	5	COSMIN
Charles 2022	2022	Early‐onset scoliosis	Diseases of the musculoskeletal system or connective tissue	81	19	1	COSMIN
Marshall 2022	2022	Sport‐related injuries	Injury, poisoning or certain other consequences of external causes	39	12	6	COSMIN
McGee 2022	2022	Endocrinology	Endocrine, nutritional or metabolic diseases	7	7	2	COSMIN
Schokman 2022	2022	Narcolepsy	Sleep‐wake disorders	19	10	2	COSMIN
Smith 2022	2022	Neurodevelopmental disability	Diseases of the nervous system	16	7	2	COSMIN
Soler 2022	2022	Neurodevelopmental disorders	Diseases of the nervous system	20	12	1	COSMIN
Mazefsky 2021	2021	Emotion regulation and reactivity	Mental, behavioral or neurodevelopmental disorders	87	87	7	Criteria set by De Los Reyes and Langer
Młyńczyk 2021	2021	Juvenile idiopathic arthritis	Diseases of the musculoskeletal system or connective tissue	41	6	4	COSMIN
Andrei 2020	2021	Excessive daytime sleepiness	Sleep‐wake disorders	16	9	1	Downs and Black checklist
GABRIELA 2021	2021	Upper‐limb function	Diseases of the musculoskeletal system or connective tissue	34	12	3	COSMIN
Mahakwe 2021	2021	Cancer	Neoplasms	19	10	10	COSMIN
Hajra 2020	2020	Negative self‐referential emotions	Mental, behavioral or neurodevelopmental disorders	103	8	2	COSMIN
Holly 2020	2020	Epilepsy	Mental, behavioral or neurodevelopmental disorders	27	11	2	COSMIN
Marson 2020	2020	Fractures	Injury, poisoning or certain other consequences of external causes	124	16	4	COSMIN
Samantha 2019	2019	Solid organ transplantation	Diseases of the immune system	62	47	1	COSMIN
Kathryn 2019	2019	Pain	Symptoms, signs or clinical findings, not elsewhere classified	80	8	3	COSMIN
Natalie 2019	2019	Mental health	Mental, behavioral or neurodevelopmental disorders	27	16	5	COSMIN
Yang 2019	2019	Oral health	Factors influencing health status or contact with health services	309	18	3	ISOQOL minimum standards
Mathews 2019	2019	Maternal/caregiver attachment	Mental, behavioral or neurodevelopmental disorders	12	6	6	COSMIN
Zaror 2019	2019	Oral health	Factors influencing health status or contact with health services	132	18	2	EMPRO tool
Stahlschmidt 2019	2019	Pain	Symptoms, signs or clinical findings, not elsewhere classified	39	12	2	COSMIN
Richard 2018	2018	Otitis media with effusion	Diseases of the ear or mastoid process	0	15	3	Modification of the criteria proposed by Andresen
Sarri 2018	2018	Sickle cell disease	Diseases of the blood or blood‐forming organs	21	24	2	COSMIN
Yolanda 2017	2017	Non‐suicidal self‐injury	Mental, behavioral or neurodevelopmental disorders	18	11	2	COSMIN
LIMPERG 2017	2017	Hemophilia	Diseases of the blood or blood‐forming organs	15	3	1	COSMIN
Dietvorst 2017	2017	Knee ligament injury	Diseases of the musculoskeletal system or connective tissue	10	6	1	COSMIN
Lucendo 2017	2017	Eosinophilic esophagitis	Diseases of the digestive system	34	3	1	COSMIN
Nobuaki 2016	2016	Cerebral palsy	Diseases of the nervous system	20	7	3	COSMIN
Ji 2016	2016	Sleep disturbances	Sleep‐wake disorders	13	6	2	COSMIN
HUSSEIN 2015	2015	Asthma	Diseases of the respiratory system	0	21	1	Combined criteria
Jennifer 2015	2015	Autism	Mental, behavioral or neurodevelopmental disorders	29	12	2	COSMIN
Myer 2015	2015	Dysphagia	Symptoms, signs or clinical findings, not elsewhere classified	5	4	4	COSMIN
SANCHEZ 2015	2015	Feeding disorders	Mental, behavioral or neurodevelopmental disorders	38	5	1	COSMIN
Alison 2014	2014	Fatigue	Factors influencing health status or contact with health services	89	20	5	COSMIN
Griffiths 2014	2014	Burn	Injury, poisoning or certain other consequences of external causes	23	32	3	COSMIN
Rainey 2014	2014	Disability (participation)	Factors influencing health status or contact with health services	22	8	1	COSMIN
Zhang 2014	2014	Chronic diseases	N/A	10	10	1	Terwee's standardized checklist
Chien 2013	2013	Disability (hand use)	Factors influencing health status or contact with health services	84	9	3	COSMIN
Paalman 2013	2013	Mental health	Mental, behavioral or neurodevelopmental disorders	29	18	3	COSMIN
Noyes 2011	2011	Health‐related quality of life and resource allocation	Factors influencing health status or contact with health services	29	4	4	COSMIN
Capio 2010	2010	Cerebral palsy	Diseases of the nervous system	12	7	2	Combined criteria
Stacey 2010	2010	Cerebral palsy	Diseases of the nervous system	8	5	2	COSMIN
Stinson 2006	2006	Pain	Symptoms, signs or clinical findings, not elsewhere classified	34	5	2	Combined criteria

Abbreviations: COSMIN, consensus‐based standards for the selection of health measurement instruments; EMPRO, evaluating measures of patient‐reported outcomes; ICD, International Classification of Diseases; ISOQOL, The International Society for Quality of Life Research; PROMs, patient‐reported outcome measures.

The included SRs addressed a total of 36 conditions, three (6.8%) SRs focused on cerebral palsy and pain, and two (4.6%) on mental health, oral health, and neurodevelopmental disorders, respectively, disability, and neurodevelopmental disorders, with disability focusing on hand use and participation, respectively. Four (9.1%) SRs addressed symptoms, including pain and fatigue, and 10 (22.7%) SRs focused on other health‐related conditions, such as mental health, oral health, emotion regulation and reactivity, or excessive daytime sleepiness (Table 1).

**FIGURE 2:**
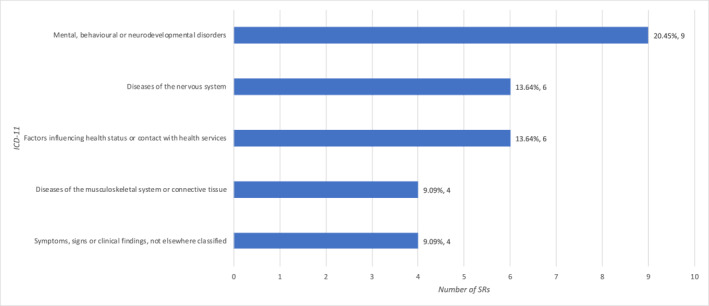
Conditions most frequently addressed by the included SRs. SRs, systematic reviews.

We used ICD‐11 to classify the 36 conditions addressed by the 44 SRs. The most frequent category was “Mental, behavioral or neurodevelopmental disorders (*n* = 9, 20.5%),” followed by “Diseases of the nervous system (6, 13.6%)” and “Factors influencing health status or contact with health services (*n* = 6, 13.6%)” (Figure 2).

### 3.2 Characteristics of recommended PROMs

The recommended PROMs were developed during the time period 1977–2019, although for 67 PROMs the development year was not reported. PROMs developed in 2012 were most frequently recommended (*n* = 5, 4.1%). The PedsQL and its subscales were the most frequently recommended, which was recommended in five disease categories (diseases of the musculoskeletal system or connective tissue, factors influencing health status or contact with health services, diseases of the immune system, diseases of the blood or blood‐forming organs and diseases of the digestive system). It was followed by the EuroQol five dimension questionnaire, which was recommended in three disease categories (sleep‐wake disorders, diseases of the musculoskeletal system or connective tissue and supplementary chapter traditional medicine conditions). The country of development was reported for 36 (29.3%) PROMs, of which two (1.6%) were developed jointly in multiple countries. The most frequent country of development was USA with 17 (13.8%) PROMs, while in the remaining countries less than five PROMs, for example, in Canada four (3.3%) and in the UK two (1.6%) (Table S3).

Forty‐two (34.2%) PROMs reported more than one validated language versions, with EuroQol Five Dimension Youth questionnaire (EQ‐5D‐Y) having the largest number of language versions, over 50. Pediatric quality of life inventory 3.0 rheumatology module (PedsQL 3.0 rheumatology module), Pediatric quality of life inventory 4.0 Generic core (PedsQL 4.0 Generic core scales), Pediatric quality of life inventory 4.0 SF15 Generic core (PedsQL 4.0 SF15 Generic core scales), on the other hand were not reported in specific language versions. Other PROMs reported in 10 or more languages are Diabetes Quality of Life for Youth Scale (*n* = 14), Behavior Rating Inventory of Executive Function (BRIEF [parent]) (*n* = 13), Early Childhood Oral Health Impact Scale (ECOHIS) (*n* = 13), Child Perceptions Questionnaire 11–14 (*n* = 12), and Duchenne muscular dystrophy Upper Limb patient‐reported outcome measures (DMD Upper Limb PROMs) (*n* = 10) (Table S3).

Only 18 (14.6%) PROMs reported completion times, and most (*n* = 12, 66.7%) PROMs could be completed in 15 min or less. The longest completion time was 30–45 min for Participation and Enjoyment/Preferences for Activities of Children (CAPE/PAC) (Table S3).

**FIGURE 3:**
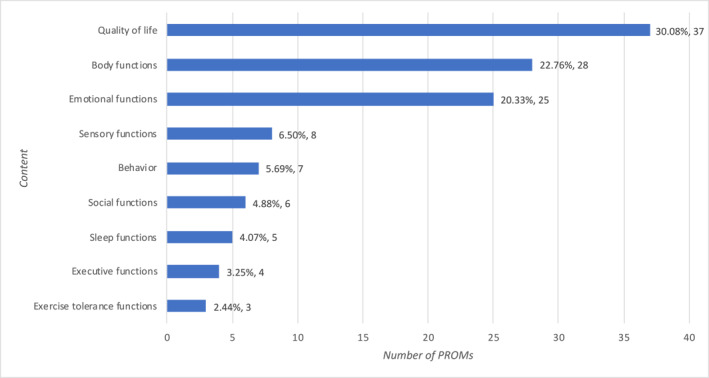
Types of content measured by the PROMs. PROMs, patient‐reported outcome measures.

Nine categories of content of interest were covered by the 123 PROMs, the most common being quality of life (*n* = 37, 30.1%), followed by body functions (*n* = 28, 22.8%) and emotional functions (*n* = 25, 20.3%; Figure 3).

**FIGURE 4:**
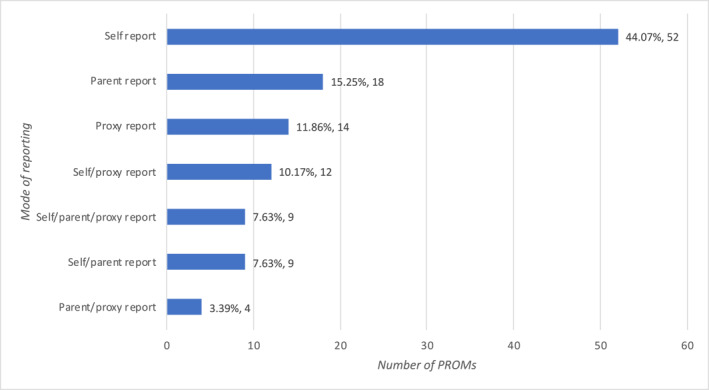
Mode of reporting of PROMs. PROMs, patient‐reported outcome measures.

Seven different modes of reporting the PROMs were identified, four of which were mixed modes: self/proxy reporting, parent/proxy reporting, self/parent reporting, and self/parent/proxy reporting. Of the 52 (44.07%) PROMs that used self reporting, 11 (21.2%) did not describe the definition of the population to which the mode was applicable; 15 (28.9%) PROMs did not specify the age of the population, although all mentioned that it was applicable to adolescents or school age children; 26 (50.0%) of the scales mentioned the age range, of which 18 (69.3%) were limited to children and adolescents over 7 years of age (Table S3 and Figure 4).

### 3.3 Measurement properties of PROMs

The majority of SRs (*n* = 35, 79.6%) used COSMIN to evaluate the measurement properties of PROMs. Three (6.8%) SRs combined multiple standards. The following six standards were adopted by one SR each: The International Society for Quality of Life Research minimum standards, Downs and Black checklist, Evaluating Measures of Patient‐Reported Outcomes tool, modification of the criteria proposed by Andresen, Terwee's standardized checklist, the criteria set by De Los Reyes and Langer (Table 1).

The measurement properties reported in the SRs are summarized in Table S4. Two measurement properties were reported in more than half of the recommended PROMs: content validity (*n* = 67, 54.5%) and internal consistency (*n* = 65, 52.9%). Measurement error had the lowest reporting rate (*n* = 10, 8.1%). The reporting rates for the remaining measurement properties were as follows: reliability (*n* = 59, 47.97%), hypotheses testing for construct validity (*n* = 57, 46.34%), structural validity (*n* = 42, 34.15%), responsiveness (*n* = 27, 21.95%), criterion validity (*n* = 20, 16.26%), and cross‐cultural validity/measurement invariance (*n* = 15, 12.20%).

### 3.4 Methodological quality of SRs

**FIGURE 5:**
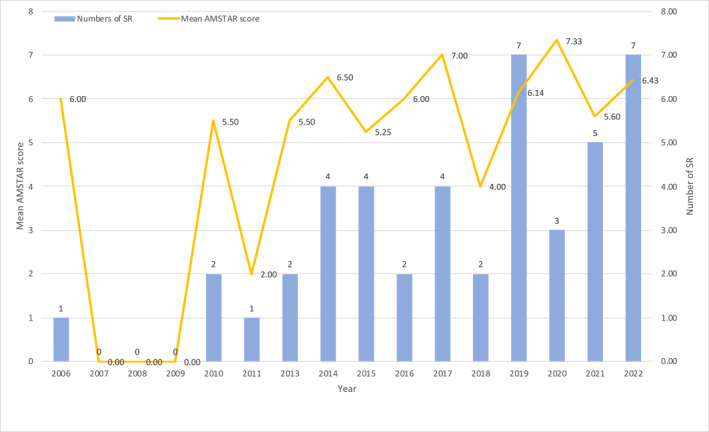
Mean AMSTAR score and number of SRs by publication year. AMSTAR, A MeaSurement Tool to Assess systematic Reviews; SRs, systematic reviews.

The mean AMSTAR score of the included SRs was 6.0 with a range of 2–9, has been fluctuating over time with no clear trend (Figure 5). Only 10 SRs (22.7%) had AMSTAR scores of 8 and above. The following four items were adhered to by at least 80% of the SR: (1) duplicate study selection and data extraction (86.4%), (2) comprehensive literature search (88.6%), (3) characteristics of the included studies (81.8%), and (4) scientific quality of the included studies (95.5%).

## 4 DISCUSSION

This study reviewed the existing SRs on pediatric PROMs developed over the last few decades and the 123 PROMs recommended in them. We found that the diversity in the target diseases and measurement content was limited. The age from which children should self‐report the measurements differed across PROMs, and some definitions were unclear. In addition, reporting of psychometric properties for SR needs improvement, and tailored tools for evaluating the methodological quality of SRs on PROMs are needed.

About half of the SRs of PROMs we identified focused on mental, behavioral or neurodevelopmental disorders, diseases of the nervous system and factors influencing health status or contact with health services; mental disorders were the most frequent topic. Quality of life, body functions and emotional functions accounted for more than 70% of the contents measured by PROMs. It indicates that more diversity in the focus diseases and the content of interest is needed. SRs synthesize and summarize existing research by evaluating the quality of studies and the consistency of results across studies and their results are considered as one of the highest levels of scientific evidence.^25^ Systematic synthesis of psychometric properties of health‐related measurement instruments is an important tool in guiding the selection of measurement instruments.^26^, ^27^ SRs of PROMs can provide pediatric clinicians and researchers the best available evidence to determine which instruments are most appropriate for use in pediatric populations in different settings, at different ages, and whether the instrument requires self‐reporting by the children or proxy reporting by caregivers or physicians.^15^ Thus, a sufficient diversity in SRs is necessary to ensure that as many pediatric diseases and specific components have an available evidence base when it comes to outcome measures.

We found that about half of the instruments were based on self‐reported measures. Some instruments used a mixed mode, requiring pediatricians to judge that whether the children's or their caregivers' reports were more trustworthy, based on the situation. We found different definitions for the age threshold between using parent/proxy reports and children's self‐reports in different PROMs. For example, the EuroQol Five Dimension Youth questionnaire (EQ‐5D‐Y) measuring the quality of life recommends children aged 8 years or above to self‐report; the Peds‐QoL 4.0 SF15 Generic core (PedsQL™ 4.0 SF15 Generic core scales) recommends already children from the age of 5 years to self‐report. Many of the PROMs intended to be self‐reported by children are designed for children aged 8 years and older,^28^ due to the belief that they have better cognitive abilities, including reading comprehension and/or auditory processing, attention, working memory, long‐term memory, time order, and judgment, to perform the mental functions required to understand items and response options, to assess and summarize their experiences related to the meaning of the items, and to select response options that best represent their self‐evaluation.^29^ However, studies that analyzed self‐reported data from 8591 children in the PedsQL^TM^ 4.0 Generic Core Scales Database^SM^ have also found that children as young as 5 years old can reliably and validly self‐report their HRQOL.^30^ Children develop at very different speeds and age alone is not necessarily sufficient to judge the child's cognitive skills. More validation testing for different age groups of children and adolescents are needed in the future and to determine the minimum age limit at which children can provide reliable and valid responses.^30^

The most important part of the SRs of PROMs is the evaluation and reporting of the measurement properties of the included PROMs, which forms the evidence base for the SRs to make recommendations on PROMs. Most of the SRs in this study used COSMIN to evaluate the measurement properties of PROMs. COSMIN is an initiative of an international multidisciplinary team of researchers with background in epidemiology, psychometrics, qualitative research, and health care.^31^ COSMIN provides not only a classification and definition of measurement properties, but also a checklist for reviewers to evaluate the risk of bias in included studies, criteria for good measurement properties, and methods for evidence synthesis.^32^ However, our study identified several problems with the reporting of measurement properties in the included SRs. First, the results of the methodological quality were not always reported simultaneously with the synthesis results. According to the COSMIN method of a best evidence synthesis, both methodological quality and consistency of results need to be synthesized. For example, strong evidence for a positive reliability is obtained when consistent positive results (ICCs or Kappa's > 0.70) are found in at least two studies of good quality or one study of excellent quality.^32^ Second, only the pooled or summary results for measurement properties were sometimes presented, without reporting an overall rating. For example, in one SR only the Cronbach's *α* = 0.76–0.93 was reported for the internal consistency of PROMs, but no overall rating (sufficient [+], insufficient [−], inconsistent [+/−], indeterminate [?]) was given.^33^ The overall rating could not be calculated from the Cronbach's *α* alone, without knowing the ratings of the individual studies. Third, the quality of evidence based on modified Grading of Recommendations Assessment, Development, and Evaluation (GRADE) approach was not always presented. COSMIN requires that the quality of evidence is graded after pooling or summarizing all evidence per measurement property and rating. Modified GRADE approach includes four factors that together determine the quality of evidence: risk of bias, inconsistency, imprecision and indirectness.^15^ Future SRs of PROMs in pediatrics should strictly follow COSMIN guidelines for evaluation, evidence synthesis, and especially reporting in the measurement properties, in order to increase the transparency of SRs.

The results of the present study show that the methodological quality of pediatric SRs on PROMs is suboptimal, and there is still room for improvement. Only four of the 11 items of the AMSTAR tool were adhered to by more than 80% of the included SRs: study selection and data extraction, comprehensive literature search, characteristics of the included studies provided, and quality of the included studies assessed. This is in line with the results of previous studies.^34^, ^35^

AMSTAR is a well‐recognized tool for evaluating the methodological quality of SRs.^22^ Both the original version and its second edition in 2017^36^ have been tested for reliability and validity. Two studies, published in 2009^26^ and 2016,^34^ have evaluated the quality of SRs of health‐related outcome measurement instruments using a custom checklist that referenced AMSTAR. The custom checklist consisted of the following items: (1) the research question, (2) the search strategy, (3) whether the inclusion and exclusion criteria were clearly described, (4) whether and how quality assessment of the included studies was performed, (5) whether and how quality assessment of the included outcome measurement instruments was performed, (6) whether and how data synthesis was performed, (7) whether article selection, data extraction, and quality assessment was done by two reviewers independently, (8) whether evidence‐based recommendations were provided, and (9) whether conflict of interest statements were included. However, the checklist was not tested for reliability and validity.^34^ We therefore decided to use AMSTAR in this study to evaluate the methodological quality of the included SRs.

The AMSTAR tool however lacks some items related to the methodology of measurement instruments, and its capability to assess the quality of SRs on such instruments was therefore limited. For example, the item on publication bias in AMSTAR is not applicable to measurement instrument studies, because of a lack of registries for these type of studies; and AMSTAR has no item to assess whether SRs provide clear recommendations for either one or multiple outcome measurement instruments. Therefore, we believe that there is a need to develop a methodological quality assessment tool for SRs of PROMs based on the COSMIN guidelines. It has also been suggested^35^ that a more comprehensive assessment of feasibility, including burden and equity, needs to be considered when the Risk of Bias tool is updated.

### 4.1 Limitations

There are several limitations to this study. First, only a limited number of common international databases and English‐language literature were searched, thus SRs published in other languages or indexed in other bibliographic databases may have been missed. Second, we extracted and analyzed only measurement properties reported for the PROMs recommended by the SRs; future analyses of other pediatrics PROMs are needed for a comprehensive understanding of the field. Third, although the SRs included in this study gave their recommended PROMs, the clinical use of these PROMs is unknown (e.g., whether the recommended PROMs are commonly used in the clinical practice). Further analysis is needed in the future for the clinical application of the recommended PROMs.

## 5 CONCLUSION

Through the descriptive analysis of existing SRs on pediatric PROMs developed over the last few decades, there is still room for improvement in both SRs and PROMs. First, more SRs of PROMs of pediatric conditions need to be focused on in order to provide more comprehensive recommendations to pediatricians. Second, more research is needed on the minimum age for self‐reporting for PROMs. Third, further research is therefore needed to determine the optimal age from which the self‐reports of children should be the primary mode of measurement reduce bias in its application. Fourth, the reporting of SRs was not standardized, the methodological quality was suboptimal, and there is in general a lack of methodological quality assessment tools suitable for SRs of PROMs. PROMs have great potential to facilitate pediatric healthcare and improve the health and lives of children and adolescents, but efforts need still to be made to help clinicians select and apply the most suitable instruments in each situation.

## AUTHOR CONTRIBUTIONS

Ruobing Lei and Yaolong Chen had full access to all the data in the study and takes responsibility for the integrity of the data and the accuracy of the data analysis. Concept and design: Yaolong Chen, Qiu Li. Acquisition, analysis, or interpretation of data: Ruobing Lei, Jin Xiong, Haiyun Wang, Yuehuan Li. Drafting of the manuscript: Ruobing Lei, Jin Xiong, Haiyun Wang. Critical revision of the manuscript for important intellectual content: Yaolong Chen, Qiu Li, Janne Estill. Statistical analysis: Ruobing Lei, Jin Xiong, Haiyun Wang. Obtained funding: Yaolong Chen, Qiu Li. Administrative, technical, or material support: Yaolong Chen, Qiu Li. Supervision: Yaolong Chen, Qiu Li.

## CONFLICT OF INTEREST STATEMENT

Yaolong Chen, Qiu Li and Janne Estill serve as editorial members of Pediatric Discovery. To minimize bias, they were excluded from all editorial decision‐making related to the acceptance of this article for publication. The authors have no other conflicts to declare.

## ETHICS STATEMENT

Not applicable.

## Supporting information

Tables S1–S4

## Data Availability

The data that supports the findings of this study are available in the supplementary material of this article.
