# Supplementary material for: Patient‐reported outcome measures in pediatrics: An overview of reviews
Source: Pediatr Discov. 2024 Jun 1;2(4):e77. doi: 10.1002/pdi3.77 (PMC12118255; doi:10.1002/pdi3.77)
Supplement: Supplementary file 1 — Tables S1–S4 [file PDI3-2-e77-s001.docx]

**eTable 1 Search strategies**

| **MEDLINE (n=1708)**   1. (((Pediatrics[MeSH Terms]) OR (Child[MeSH Terms])) OR ("Infant"[Mesh])) OR (((((((((((((((((((((((pediatric*[Title]) OR (paediatric*[Title])) OR (child*[Title])) OR (infant*[Title])) OR (adolescent*[Title])) OR (baby[Title])) OR (babies[Title])) OR (trottie*[Title])) OR (youth*[Title])) OR (kids[Title])) OR (toddler*[Title])) OR (pre-school*[Title])) OR (preschool*[Title])) OR (junior*[Title])) OR (juvenile*[Title])) OR (neonat*[Title])) OR (newborn*[Title])) OR (teenager*[Title])) OR (pubescent[Title])) OR (preterm[Title])) OR (puberty[Title])) OR (young*[Title]))) 2. “Quality of Life”[MeSH] OR "Patient Reported Outcome Measures"[Mesh] OR "Patient Outcome assessment"[Mesh:noexp] OR "Surveys and questionnaires"[Mesh:noexp] OR “Self report”[MeSH] OR HR-PRO[tiab] OR HRPRO[tiab] OR HRQL[tiab] OR HRQoL[tiab] OR QL[tiab] OR QoL[tiab] OR quality of life[tiab] OR life quality[tiab] OR health index*[tiab] OR health indices[tiab] OR health profile*[tiab] OR health status[tiab] 3. (instrumentation[sh] OR methods[sh] OR Validation Study[pt] OR Comparative Study[pt] OR “psychometrics”[MeSH] OR psychometr*[tiab] OR clinimetr*[tw] OR clinometr*[tw] OR "Outcome Assessment, Health Care"[Mesh] OR outcome assessment[tiab] OR outcome measure*[tw] OR “observer variation”[MeSH] OR observer variation[tiab] OR “Health Status Indicators”[Mesh] OR “reproducibility of results”[MeSH] OR reproducib*[tiab] OR “discriminant analysis”[MeSH] OR reliab*[tiab] OR unreliab*[tiab] OR valid*[tiab] OR coefficient[tiab] OR homogeneity[tiab] OR homogeneous[tiab] OR “internal consistency”[tiab] OR (cronbach*[tiab] AND (alpha[tiab] OR alphas[tiab])) OR (item[tiab] AND (correlation*[tiab] OR selection*[tiab] OR reduction*[tiab])) OR agreement[tiab] OR precision[tiab] OR imprecision[tiab] OR “precise values”[tiab] OR test–retest[tiab] OR (test[tiab] AND retest[tiab]) OR (reliab*[tiab] AND (test[tiab] OR retest[tiab])) OR stability[tiab] OR interrater[tiab] OR inter-rater[tiab] OR intrarater[tiab] OR intra-rater[tiab] OR intertester[tiab] OR inter-tester[tiab] OR intratester[tiab] OR intra-tester[tiab] OR interobserver[tiab] OR inter-observer[tiab] OR intraobserver[tiab] OR intra-observer[tiab] OR intertechnician[tiab] OR inter-technician[tiab] OR intratechnician[tiab] OR intra-technician[tiab] OR interexaminer[tiab] OR inter-examiner[tiab] OR intraexaminer[tiab] OR intra-examiner[tiab] OR interassay[tiab] OR inter-assay[tiab] OR intraassay[tiab] OR intra-assay[tiab] OR interindividual[tiab] OR inter-individual[tiab] OR intraindividual[tiab] OR intra-individual[tiab] OR interparticipant[tiab] OR inter-participant[tiab] OR intraparticipant[tiab] OR intra-participant[tiab] OR kappa[tiab] OR kappa’s[tiab] OR kappas[tiab] OR repeatab*[tiab] OR ((replicab*[tiab] OR repeated[tiab]) AND (measure[tiab] OR measures[tiab] OR findings[tiab] OR result[tiab] OR results[tiab] OR test[tiab] OR tests[tiab])) OR generaliza*[tiab] OR generalisa*[tiab] OR concordance[tiab] OR (intraclass[tiab] AND correlation*[tiab]) OR discriminative[tiab] OR “known group”[tiab] OR factor analysis[tiab] OR factor analyses[tiab] OR dimension*[tiab] OR subscale*[tiab] OR (multitrait[tiab] AND scaling[tiab] AND (analysis[tiab] OR analyses[tiab])) OR item discriminant[tiab] OR interscale correlation*[tiab] OR error[tiab] OR errors[tiab] OR “individual variability”[tiab] OR (variability[tiab] AND (analysis[tiab] OR values[tiab])) OR (uncertainty[tiab] AND (measurement[tiab] OR measuring[tiab])) OR “standard error of measurement”[tiab] OR sensitiv*[tiab] OR responsive*[tiab] OR ((minimal[tiab] OR minimally[tiab] OR clinical[tiab] OR clinically[tiab]) AND (important[tiab] OR significant[tiab] OR detectable[tiab]) AND (change[tiab] OR difference[tiab])) OR (small*[tiab] AND (real[tiab] OR detectable[tiab]) AND (change[tiab] OR difference[tiab])) OR meaningful change[tiab] OR “ceiling effect”[tiab] OR “floor effect”[tiab] OR “Item response model”[tiab] OR IRT[tiab] OR Rasch[tiab] OR “Differential item functioning”[tiab] OR DIF[tiab] OR “computer adaptive testing”[tiab] OR “item bank”[tiab] OR “cross-cultural equivalence”[tiab]) 4. “address”[Publication Type] OR “biography”[Publication Type] OR “case reports”[Publication Type] OR “comment”[Publication Type] OR “directory”[Publication Type] OR “editorial”[Publication Type] OR “festschrift”[Publication Type] OR “interview”[Publication Type] OR “lecture”[Publication Type] OR “legal case”[Publication Type] OR “legislation”[Publication Type] OR “letter”[Publication Type] OR “news”[Publication Type] OR “newspaper article”[Publication Type] OR “patient education handout”[Publication Type] OR “popular work”[Publication Type] OR “congress”[Publication Type] OR “consensus development conference”[Publication Type] OR “consensus development conference, nih”[Publication Type] OR “practice guideline”[Publication Type] NOT (“animals”[MeSH Terms] NOT “humans”[MeSH Terms]) 5. #1 AND #2 AND #3 NOT #4 6. (((systematic review[Title] OR systematic literature review[Title] OR systematic scoping review[Title] OR systematic narrative review[Title] OR systematic qualitative review[Title] OR systematic evidence review[Title] OR systematic quantitative review[Title] OR systematic meta-review[Title] OR systematic critical review[Title] OR systematic mixed studies review[Title] OR systematic mapping review[Title] OR systematic cochrane review[Title] OR systematic search and review[Title] OR systematic integrative review[Title]) NOT comment[Publication type] NOT (protocol[Title] OR protocols[Title])) NOT MEDLINE [subset]) OR (Cochrane Database Syst Rev[ta] AND review[Publication type]) OR systematic review[Publication type] OR Systematic Reviews as Topic [Mesh] 7. #5 AND #6 |
| --- |
| **Embase (n=2090)**   1. 'adolescent'/exp 2. 'child'/exp 3. 'pediatrics'/exp 4. 'adolescen*':ab,ti 5. 'teen*':ab,ti 6. 'youth*':ab,ti 7. 'juvenile*':ab,ti 8. 'puberty':ab,ti 9. 'young*':ab,ti 10. 'child*':ab,ti 11. 'pediatric*':ab,ti 12. 'paediatric*':ab,ti 13. 'infant*':ab,ti 14. 'neonat*':ab,ti 15. 'newborn*':ab,ti 16. 'baby':ab,ti 17. 'babies':ab,ti 18. 'toddler*':ab,ti 19. 'pre-school*':ab,ti 20. 'preschool*':ab,ti 21. 'junior*':ab,ti 22. 'pubescent':ab,ti 23. OR #1-#22 24. 'quality of life'/exp OR 'patient-reported outcome'/mj OR 'questionnaire'/mj OR 'self report'/mj 25. (HRPRO or HRQL or HRQoL or QL or QoL or quality of life or life quality or health index* or health indices or health profile* or health status):ti,ab 26. #24 OR #25 27. 'psychometry'/exp or 'outcome assessment'/exp or 'validation study'/exp or 'observer variation'/exp or 'health status indicator'/exp or 'reproducibility'/exp or 'discriminant analysis'/exp 28. (psychometr* or clinimetr* or clinometr* or 'outcome assessment' or 'outcome measure*' or 'observer variation' or reproducib* or reliab* or unreliab* or valid* or 'coefficient of variation' or coefficient or homogeneity or homogeneous or 'internal consistency'):ti,ab 29. cronbach*:ti,ab and (alpha or alphas):ti,ab 30. Item:ti,ab and (correlation* or selection* or reduction*):ti,ab 31. (agreement or precision or imprecision or 'precise values' or test-retest):ti,ab 32. (test and retest):ti,ab 33. (reliab*:ti,ab and (test or retest)):ti,ab 34. (stability or interrater or inter-rater or intrarater or intra-rater or intertester or inter-tester or intratester or intra-tester or interobserver or inter-observer or intraobserver or intra-observer or intertechnician or inter-technician or intratechnician or intra-technician or interexaminer or inter-examiner or intraexaminer or intra-examiner or interassay or inter-assay or intraassay or intra-assay or interindividual or inter-individual or intraindividual or intra-individual or interparticipant or inter-participant or intraparticipant or intraparticipant or kappa* or repeatab*):ti,ab 35. (replicab* or repeated):ti,ab and (measure or measures or findings or result or results or test or tests):ti,ab 36. (generaliza* or generalisa* or concordance):ti,ab 37. (intraclass and correlation*):ti,ab 38. (discriminative or 'known group' or 'factor analysis' or 'factor analyses' or 'factor structure' or 'factor structures' or dimension* or subscale*):ti,ab 39. multitrait:ti,ab and scaling:ti,ab and (analysis or analyses):ti,ab 40. ('item discriminant' or 'interscale correlation*' or error or errors or 'individual variability' or 'interval variability' or 'rate variability'):ti,ab 41. Variability:ti,ab and (analysis or values):ti,ab 42. uncertainty and (measurement or measuring):ti,ab 43. ('standard error of measurement' or sensitiv* or responsive*):ti,ab 44. ('minimal detectable concentration' or interpretab*):ti,ab 45. (minimal or minimally or clinical or clinically):ti,ab and (important or significant or detectable):ti,ab and (change or difference):ti,ab 46. small*:ti,ab and (real or detectable):ti,ab and (change or difference):ti,ab 47. ('meaningful change' or 'ceiling effect' or 'floor effect' or 'Item response model' or IRT or Rasch or 'Differential item functioning' or DIF or 'computer adaptive testing' or 'item bank' or 'cross-cultural equivalence'):ti,ab 48. OR #27-#47 49. 'meta analysis'/exp OR 'systematic review'/exp OR (meta NEAR/3 analy*):ab,ti OR metaanaly*:ab,ti OR review*:ti OR overview*:ti OR (synthes* NEAR/3 (literature* OR research* OR studies OR data)):ab,ti OR (pooled AND analys*:ab,ti) OR ((data NEAR/2 pool*):ab,ti AND studies:ab,ti) OR medline:ab,ti OR medlars:ab,ti OR embase:ab,ti OR cinahl:ab,ti OR scisearch:ab,ti OR psychinfo:ab,ti OR psycinfo:ab,ti OR psychlit:ab,ti OR psyclit:ab,ti OR cinhal:ab,ti OR cancerlit:ab,ti OR cochrane:ab,ti OR bids:ab,ti OR pubmed:ab,ti OR ovid:ab,ti OR ((hand OR manual OR database* OR computer*) NEAR/2 search*):ab,ti OR (electronic NEAR/2 (database* OR 'data base' OR 'data bases')):ab,ti OR bibliograph*:ab OR 'relevant journals':ab OR ((review* OR overview*) NEAR/10 (systematic* OR methodologic* OR quantitativ* OR research* OR literature* OR studies OR trial* OR effective*)):ab NOT (((retrospective* OR record* OR case* OR patient*) NEAR/2 review*):ab,ti OR ((patient* OR review*) NEAR/2 chart*):ab,ti OR rat:ab,ti OR rats:ab,ti OR mouse:ab,ti OR mice:ab,ti OR hamster:ab,ti OR hamsters:ab,ti OR animal:ab,ti OR animals:ab,ti OR dog:ab,ti OR dogs:ab,ti OR cat:ab,ti OR cats:ab,ti OR bovine:ab,ti OR sheep:ab,ti) NOT ('editorial'/exp OR 'erratum'/de OR 'letter'/exp) NOT ('animal'/exp OR 'nonhuman'/exp NOT ('animal'/exp OR 'nonhuman'/exp AND 'human'/exp)) 50. #23 AND #26 AND #48 AND #49 |
| **Cochrane (n=1758)**   1. paediatric*:ti,ab,kw OR pediatric*:ti,ab,kw 2. child*:ti,ab,kw 3. bab*:ti,ab,kw 4. infan*:ti,ab,kw 5. toddler*:ti,ab,kw 6. preschool:ti,ab,kw OR pre-school:ti,ab,kw 7. adolescen*:ti,ab,kw 8. youth*:ti,ab,kw 9. teenage*:ti,ab,kw 10. MeSH descriptor: [Child] this term only 11. MeSH descriptor: [Pediatrics] this term only 12. MeSH descriptor: [Infant] this term only 13. MeSH descriptor: [Adolescent] this term only 14. OR #1-#13 15. MeSH descriptor: [Quality of Life] explode all trees 16. MeSH descriptor: [Patient Reported Outcome Measures] explode all trees 17. MeSH descriptor: [Patient Outcome Assessment] explode all trees 18. MeSH descriptor: [Surveys and Questionnaires] explode all trees 19. MeSH descriptor: [Self Report] explode all trees 20. (HRPRO or HRQL or HRQoL or QL or QoL or quality of life or life quality or health index* or health indices or health profile* or health status):ti,ab,kw or ((patient or self or proxy):ti,ab,kw and ((report or reported or reporting) or (rated or rating or ratings):ti,ab,kw or based:ti,ab,kw or (assessed or assessment or assessments))):ti,ab,kw or ((disability or function or functional or functions or subjective or utility or utilities or wellbeing or well being) and (index or indices or instrument or instruments or measure or measures or questionnaire or questionnaires or profile or profiles or scale or scales or score or scores or status or survey or surveys)):ti,ab,kw 21. OR #15-#20 22. MeSH descriptor: [Psychometrics] explode all trees 23. MeSH descriptor: [Outcome Assessment, Health Care] explode all trees 24. MeSH descriptor: [Observer Variation] explode all trees 25. MeSH descriptor: [Health Status Indicators] explode all trees 26. MeSH descriptor: [Discriminant Analysis] explode all trees 27. (psychometr* or clinimetr* or clinometr* or 'outcome assessment' or 'outcome measure*' or 'observer variation' or reproducib* or reliab* or unreliab* or valid* or 'coefficient of variation' or coefficient or homogeneity or homogeneous or 'internal consistency'):ti,ab,kw 28. cronbach*:ti,ab,kw and (alpha or alphas):ti,ab,kw 29. Item:ti,ab,kw and (correlation* or selection* or reduction*):ti,ab,kw 30. (agreement or precision or imprecision or 'precise values' or test-retest):ti,ab,kw 31. (test and retest):ti,ab,kw 32. (reliab*:ti,ab,kw and (test or retest)):ti,ab,kw 33. (stability or interrater or inter-rater or intrarater or intra-rater or intertester or inter-tester or intratester or intra-tester or interobserver or inter-observer or intraobserver or intra-observer or intertechnician or inter-technician or intratechnician or intra-technician or interexaminer or inter-examiner or intraexaminer or intra-examiner or interassay or inter-assay or intraassay or intra-assay or interindividual or inter-individual or intraindividual or intra-individual or interparticipant or inter-participant or intraparticipant or intraparticipant or kappa* or repeatab*):ti,ab,kw 34. (replicab* or repeated):ti,ab,kw and (measure or measures or findings or result or results or test or tests):ti,ab,kw 35. (generaliza* or generalisa* or concordance):ti,ab,kw 36. (intraclass and correlation*):ti,ab,kw 37. (discriminative or 'known group' or 'factor analysis' or 'factor analyses' or 'factor structure' or 'factor structures' or dimension* or subscale*):ti,ab,kw 38. multitrait:ti,ab,kw and scaling:ti,ab,kw and (analysis or analyses):ti,ab,kw 39. ('item discriminant' or 'interscale correlation*' or error or errors or 'individual variability' or 'interval variability' or 'rate variability'):ti,ab,kw 40. Variability:ti,ab,kw and (analysis or values):ti,ab,kw 41. uncertainty and (measurement or measuring):ti,ab,kw 42. ('standard error of measurement' or sensitiv* or responsive*):ti,ab,kw 43. ('minimal detectable concentration' or interpretab*):ti,ab,kw 44. (minimal or minimally or clinical or clinically):ti,ab,kw and (important or significant or detectable):ti,ab,kw and (change or difference):ti,ab,kw 45. small*:ti,ab,kw and (real or detectable):ti,ab,kw and (change or difference):ti,ab,kw 46. ('meaningful change' or 'ceiling effect' or 'floor effect' or 'Item response model' or IRT or Rasch or 'Differential item functioning' or DIF or 'computer adaptive testing' or 'item bank' or 'cross-cultural equivalence'):ti,ab,kw 47. OR #22-#46 48. #14 AND #21 AND #47 |

**eTable 2 List of excluded studies**

| **Title of excluded study** | **Reason for exclusion** |
| --- | --- |
| Approaches to Measure Sleep-Wake Disturbances in Adolescents with Cancer | Not SR of PROMs |
| Assessing Patient-Reported Outcomes in Pediatric Populations With Vaccine-Preventable Infectious Diseases: A Systematic Review of the Literature (the PROCHID Study) | Without recommendations |
| Assessing severity of illness and outcomes of treatment in children with Chronic Fatigue Syndrome/Myalgic Encephalomyelitis (CFS/ME): a systematic review of patient-reported outcome measures (PROMs) | Without recommendations |
| Assessment of Behavioral and Emotional Problems in Infancy: A Systematic Review | Not SR of PROMs |
| Assessment of child’s dental anxiety/fear and stress during dental treatment: a systematic review by CEDACORE | Without measurement properties |
| Assessment of quality of life in children, adolescents, and adults with celiac disease through specific questionnaires: Review | Without recommendations |
| Assessment of stigma related to visible skin diseases: a systematic review and evaluation of patient-reported outcome measures | Unable to extract pediatric PROM data |
| Assessments of sensory processing in infants: a systematic review | Without recommendations |
| Autism Screening Tests A Narrative Review | Without measurement properties |
| Best practices for assessing environmental tobacco smoke exposure in children using questionnaires: A systematic review of measurement properties | Not SR of PROMs |
| Can we rely on adolescents to self-assess puberty stage? A systematic review and meta-analysis | Without measurement properties |
| Considering quality of life for children with cancer: a systematic review of patient-reported outcome measures and the development of a conceptual model | Without measurement properties |
| Diagnostic accuracy of screening questionnaires for obstructive sleep apnea in children: A systematic review and meta-analysis | Without measurement properties |
| Different Domains of Dengue Research in Malaysia: A Systematic Review and Meta-Analysis of Questionnaire-Based Studies | Unable to extract pediatric PROM data |
| Disease-Specific Outcome Measures Evaluating the Health-Related Quality of Life of Children and Adolescents with Idiopathic Scoliosis and Scheuermann’s Kyphosis: A Literature Review | Without recommendations |
| Do parents and children agree on rating a child’s HRQOL? A systematic review and Meta-analysis of comparisons between children with attention deficit hyperactivity disorder and children with typical development using the PedsQL | Without measurement properties |
| Health-related quality of life questionnaires in individuals with haemophilia: a systematic review of their measurement properties | Without recommendations |
| Health-related quality of life and functional outcome measures for pediatric multiple injury: A systematic review and narrative synthesis | Without recommendations |
| Health-related quality-of-life outcome measures in paediatric palliative care: A systematic review of psychometric properties and feasibility of use | Without recommendations |
| How Accurate is Subjective Reporting of Childhood Sleep Patterns? A Review of the Literature and Implications for Practice | Without recommendations |
| Identifying eating disorders in adolescents and adults with overweight or obesity: A systematic review of screening questionnaires | Without measurement properties |
| Impact of using patient-reported outcome measures in routine clinical care of paediatric patients with chronic conditions: a systematic review protocol | Not SR of PROMs |
| Instruments for the Assessment of Behavioral and Psychosocial Functioning in Duchenne and Becker Muscular Dystrophy; a Systematic Review of the Literature | Unable to extract pediatric PROM data |
| Instruments Measuring Self-Care in Children and Young Adults With Chronic Conditions: A Systematic Review | Unable to extract pediatric PROM data |
| Instruments to measure anxiety in children, adolescents, and young adults with cancer: a systematic review | Without recommendations |
| Is there a difference between child self-ratings and parent proxy- ratings of the quality of life of children with a diagnosis of attention-deficit hyperactivity disorder (ADHD)? A systematic review of the literature | Without recommendations |
| The Majority of Patient-reported Outcome Measures in Pediatric Orthopaedic Research Are Used Without Validation | Without measurement properties |
| The measurement of health-related quality of life (QOL) in paediatric clinical trials: a systematic review | Without measurement properties |
| Measurement of health‑related quality of life in pediatric organ transplantation recipients: a systematic review of the PedsQL transplant module | Not SR of PROMs |
| Measurement properties of quality of life measurement instruments for infants, children and adolescents with eczema: protocol for a systematic review | Not SR of PROMs |
| Measurement properties of quality‐of‐life outcome measures for children and adults with eczema: An updated systematic review | Without recommendations |
| Measurement tools for gender identity, gender expression, and gender dysphoria in transgender and gender-diverse children and adolescents: a systematic review | Without measurement properties |
| "Measurements of quality of life for children with cerebral palsy." Archives of Disease in Childhood | Not SR of PROMs |
| Measuring health-related quality of life after pediatric cochlear implantation: A systematic review | Without measurement properties |
| Measuring quality of life in children with speech and language difficulties: a systematic review of existing approaches | Without measurement properties |
| Measuring What Matters for Children: A Systematic Review of Frequently Used Pediatric Generic PRO Instruments | Without measurement properties |
| The Neonatal Behavioral Assessment Scale (NBAS) and Newborn Behavioral Observations (NBO) system for supporting caregivers and improving outcomes in caregivers and their infants (Review) | Not SR of PROMs |
| Oral Health-Related Quality of Life in Adolescents as Measured with the Child-OIDP Questionnaire: A Systematic Review | Without measurement properties |
| Outcome measures for assessing change over time in studies of symptomatic children with hypermobility: a systematic review | Not SR of PROMs |
| Patient- and parent proxy-reported outcome measures for life participation in children with chronic kidney disease: a systematic review | Without recommendations |
| Patient-Reported Outcome Measures Following Surgical Intervention for Pediatric Sports-Related Injuries to the Knee: a Systematic Review | Without measurement properties |
| Patient-Reported Outcome Measures for Adults and Adolescents with Patellofemoral Pain: A Systematic Review of Content Validity and Feasibility Using the COSMIN Methodology | Unable to extract pediatric PROM data |
| Patient-Reported Outcome Measures for Use in Clinical Trials and Clinical Practice in Inflammatory Bowel Diseases: A Systematic Review | Unable to extract pediatric PROM data |
| Patient-reported Outcome Measures in Pediatric Non- Malignant Hematology: A Systematic Review | Without measurement properties |
| Patient-reported outcome measures in pediatric palliative care—a protocol for a scoping review | Not SR of PROMs |
| Patient-reported outcome measures in pediatric surgery - A systematic review | Not SR of PROMs |
| patient-reported outcomes” in paediatric oncology: Overview, clarifications and articulations. Towards mutual comprehension and shared objectives | Not SR of PROMs |
| Pediatric Quality of Life Instruments in Oral Health Research: A Systematic Review | Without measurement properties |
| Physical Activity Questionnaires for Youth A Systematic Review of Measurement Properties | Without recommendations |
| Physical Functioning in Adolescents with Idiopathic Scoliosis | Without recommendations |
| A comparison of self-reported and proxy-reported health utilities in children: a systematic review and meta-analysis | Without recommendations |
| Preference-based measures of health-related quality of life in congenital mobility impairment: a systematic review of validity and responsiveness | Unable to extract pediatric PROM data |
| Protocol for a systematic review exploring the psychometric properties of self-report health-related quality of life and subjective wellbeing measures used by adolescents with intellectual disabilities | Not SR of PROMs |
| Psychometric properties of gross motor assessment tools for children: a systematic review | Without recommendations |
| Psychometric Properties of the Numerical RatingScale to Assess Self-Reported Pain Intensity in Children and Adolescents | Unable to extract pediatric PROM data |
| Quality of health literacy instruments used in children and adolescents: a systematic review | Without recommendations |
| Quality of life and exercise tolerance tools in children/adolescents with cystic fibrosis: Systematic review | Without recommendations |
| Quality of life in asthmatic children: A literature review. | Without recommendations |
| Questionnaires assessing the use of complementary health approaches in pediatrics and their measurement properties: A systematic review | Without recommendations |
| Questionnaires for the Measurement of Infant Environmental Tobacco Smoke Exposure: A Systematic Review | Without recommendations |
| Questionnaires Measuring Physical Activity in Clinical Pediatric Populations: A Systematic Review | Without recommendations |
| Reliability and Validity of Child/Adolescent Food Frequency Questionnaires That Assess Foods and/or Food Groups | Without recommendations |
| Reliability of generic quality-of-life instruments in assessing health-related quality of life among children and adolescents with idiopathic nephrotic syndrome: a systematic review | Without measurement properties |
| Reliability of Instruments Measuring At-Risk and Problem Gambling Among Young Individuals: A Systematic Review Covering Years 2009e2015 | Unable to extract pediatric PROM data |
| Review of life satisfaction measures for adolescents. | Not SR of PROMs |
| A Review of Scales to evaluate Sleep Disturbances in Movement Disorders | Unable to extract pediatric PROM data |
| Self-report use-of-time tools for the assessment of physical activity and sedentary behaviour in young people: systematic review | Without recommendations |
| Self-Reported Quality of Life of Young Children with Conditions from Early Infancy: A Systematic Review | Without recommendations |
| Short Tools to Assess Young Children’s Dietary Intake: A Systematic Review Focusing on Application to Dietary Index Research | Without recommendations |
| Sleep quality in children: questionnaires available in Brazil | Without measurement properties |
| Systematic review and critical appraisal of Childhood Trauma Questionnaire — Short Form (CTQ-SF) | Not SR of PROMs |
| Systematic Review of Childhood Sedentary Behavior Questionnaires: What do We Know and What is Next? | Without recommendations |
| Systematic Review of Conceptual, Age, Measurement and Valuation Considerations for Generic Multidimensional Childhood Patient‑Reported Outcome Measures | Without recommendations |
| A Systematic Review of Core Outcomes for Hypospadias Surgery | Unable to extract pediatric PROM data |
| A systematic review of measures of activity limitation for children with cerebral palsy | Without recommendations |
| A Systematic Review of Measures of Breakthrough Pain and Their Psychometric PropertiesTaggedEnd | Unable to extract pediatric PROM data |
| A systematic review of patient-reported outcome measures of neuropathy in children, adolescents and young adults | Unable to extract pediatric PROM data |
| A systematic review of proxy-report questionnaires assessing physical activity, sedentary behavior and/or sleep in young children (aged 0–5 years) | Without recommendations |
| Systematic review of self-concept measures for primary school aged children with cerebral palsy | Without recommendations |
| A systematic review of symptom assessment scales in children with cancer | Without recommendations |
| A Systematic Review of the Psychometric Properties of Bronchiolitis Assessment Tools | Unable to extract pediatric PROM data |
| Systematic Review of Tools and Methods to Measure Appetite in Undernourished Children in the Context of Low- and Middle-Income Countries | Without recommendations |
| A Systematic Review of Trauma Screening Measures for Children and Adolescents | Without recommendations |
| Systematic review: measurement properties of patient- reported outcome measures evaluated with childhood brain tumor survivors or other acquired brain injury | Without recommendations |
| Systematic Review: Non-Instrumental Swallowing and Feeding Assessments in Pediatrics | Not SR of PROMs |
| An Updated Systematic Review of Childhood Physical Activity Questionnaires | Without recommendations |
| [Validity and reliability of self-report instruments for measuring physical activity in adolescents: a systematic review] | Without recommendations |
| Validity of short food questionnaire items to measure intake in children and adolescents: a systematic review | Without recommendations |
| What is the Validity of Questionnaires Assessing Fruit and V egetable Consumption in Children when Compared with Blood Biomarkers? A Meta-Analysis | Without recommendations |
| Quality of life in paediatric gastrointestinal and liver disease: a systematic review | Unable to extract pediatric PROM data |
| Subjective versus Objective Measure of Physical Activity: A Systematic Review and Meta-Analysis of the Convergent Validity of the Physical Activity Questionnaire for Children (PAQ-C) | Without measurement properties |
| [Physical activity questionnaires for Spanish children and adolescents: a systematic review] | Unable to extract pediatric PROM data |
| Subjective reports of children's sleep duration: does the question matter? A literature review | Unable to extract pediatric PROM data |
| Measuring patient-reported mental health outcomes in youth with neurodevelopmental disorders: a scoping review | Unable to extract pediatric PROM data |
| Use of the child health questionnaire in children with cerebral palsy: a systematic review and evaluation of the psychometric properties | Unable to extract pediatric PROM data |
| Quality of life in survivors of childhood cancer: a systematic review of the literature (2001-2008) | Without measurement properties |
| Assessing the engagement of children and families in selecting patient-reported outcomes (PROs) and developing their measures (PROMs) | Unable to extract pediatric PROM data |
| Assessing the engagement of children and families in selecting patient-reported outcomes (PROs) and developing their measures: a systematic review | Not SR of PROMs |
| A systematic review of clinimetric properties of measurements of motivation for children aged 5-16 years with a physical disability or motor delay | Unable to extract pediatric PROM data |
| Measurement of habitual physical activity in primary school-aged children with cerebral palsy: A systematic review | Without recommendations |
| Quality of life measures used in radiation therapy trials for patients with metastatic spinal cord compression (MSCC): A literature review | Unable to extract pediatric PROM data |
| Patient-reported outcomes in rehabilitation research: Instruments and current developments in Germany | Without measurement properties |
| Pediatric patient-reported outcomes assessment: A case study in epidermolysis bullosa | Unable to extract pediatric PROM data |
| Assessment of health-related quality of Life (HRQoL) measures for paediatric patients with anaemia of chronic kidney disease (CKD) | Unable to extract pediatric PROM data |
| Psychometric Properties of Assessments of Cognition in Infants With Cerebral Palsy or Motor Impairment: A Systematic Review | Unable to extract pediatric PROM data |
| A Systematic Review of International Guidance for Self-Report and Proxy Completion of Child-Specific Utility Instruments | Without measurement properties |
| Protocol for a systematic review of instruments for the assessment of quality of life and well-being in children and adolescents with cerebral palsy | Unable to extract pediatric PROM data |
| A review of preference-based measures for the assessment of quality of life in children and adolescents with cerebral palsy | Unable to extract pediatric PROM data |
| Validated instruments for measuring the oral health in children | Unable to extract pediatric PROM data |
| Utility of the Ages and Stages Questionnaire to Identify Developmental Delay in Children Aged 12 to 60 Months: A Systematic Review and Meta-analysis | Without measurement properties |
| Psychometric properties of instruments for assessing depression among African youth: A systematic review | Unable to extract pediatric PROM data |
| Pediatric dysphagia assessment tools: A systematic review | Unable to extract pediatric PROM data |
| Validity and reliability of sleep time questionnaires in children and adolescents: A systematic review and meta-analysis | Unable to extract pediatric PROM data |
| Impact of methodological approaches in the agreement between subjective and objective methods for assessing screen time and sedentary behavior in pediatric population: a systematic review | Not SR of PROMs |
| Health-related quality of life in paediatric patients with vitiligo: A systematic review and meta-analysis | Without measurement properties |
| Health-related quality of life of young adult survivors of childhood cancer: A review of qualitative studies | Not SR of PROMs |
| Generic health literacy measurement instruments for children and adolescents: a systematic review of the literature | Unable to extract pediatric PROM data |
| Validity and Reliability of Questionnaires That Assess Barriers and Facilitators of Sedentary Behavior in the Pediatric Population: A Systematic Review | Unable to extract pediatric PROM data |
| Subsidising artemisinin‐based combination therapy in the private retail sector | Without measurement properties |
| Measuring mental wellness among adolescents living with a physical chronic condition: a systematic review of the mental health and mental well-being instruments | Without measurement properties |
| The usefulness of personal risk questionnaires for identifying lead-poisoned children | Unable to extract pediatric PROM data |
| A systematic review of screening questionnaires for childhood lead poisoning | Without measurement properties |
| The Adverse Childhood Experiences - International Questionnaire (ACE-IQ) in community samples around the world: A systematic review (part I) | Without measurement properties |
| International Comparison of Self-Concept, Self-Perception and Lifestyle in Adolescents: A Systematic Review | Without measurement properties |
| Health-related quality of life after pediatric liver transplantation: A systematic review | Without measurement properties |
| Child and adolescent self-report symptom measurement in pediatric oncology research: a systematic literature review | Unable to extract pediatric PROM data |
| Is Transcatheter Aortic Valve Implantation (TAVI) Associated With Improvement in Quality of Life? A Systematic Review | Unable to extract pediatric PROM data |
| Measurement of sedentary behaviour in population health surveys: A review and recommendations | Without measurement properties |
| Measuring health-related quality of life for child maltreatment: a systematic literature review | Without measurement properties |
| Instruments to measure health-related quality of life in children and adolescents with inflammatory bowel disease - Systematic review and qualitative analysis | Unable to extract pediatric PROM data |
| Child Attitude Toward Illness Scale (CATIS): A systematic review of the literature | Without measurement properties |
| Patient-reported outcomes following cleft surgery: A systematic review | Unable to extract pediatric PROM data |
| Systematic review on measurement properties of questionnaires assessing the neighbourhood environment in the context of youth physical activity behaviour | Unable to extract pediatric PROM data |
| Pharmacological treatment in functional abdominal pain disorders in children: A systematic review and meta-analysis | Unable to extract pediatric PROM data |
| Questionnaires Measuring 24-Hour Movement Behaviors in Childhood and Adolescence: Content Description and Measurement Properties-A Systematic Review | Without recommendations |
| A review of the characteristics of validated quality of life (QOL) patient reported outcome measures (PROMs) in paediatric plastic surgery | Unable to extract pediatric PROM data |
| Do we measure the right thing? -Lack of evidence for the content validity of health-related quality-of-life questionnaires for children with cancer | Unable to extract pediatric PROM data |
| What Matters to Children with Cancer a Systematic Review of Qualitative Studies and Patient-Reported Outcome Measures (PROMS) | Unable to extract pediatric PROM data |
| Patient-reported outcome measures in children with cystic fibrosis | Unable to extract pediatric PROM data |
| Identifying mental health needs of children and youth with skin disease: A systematic review of screening and assessment tools | Without recommendations |
| Assessing the conceptual content in health status and quality of life instruments in paediatric epilepsy using World Health Organization definitions | Unable to extract pediatric PROM data |
| Assessing the conceptual content in health status and quality of life measures in pediatric epilepsy using world health organization definitions | Unable to extract pediatric PROM data |
| Evaluation of psychometric properties of patient-reported outcome measures in pediatric psoriasis | Unable to extract pediatric PROM data |
| Patient- and proxy-reported outcome measures instruments for the assessment of asthma control among adult and pediatric population: A protocol for systematic review | Not SR of PROMs |
| Quality of patient-reported and proxy-reported outcomes for children with impairment of the lower extremity: systematic review protocol | Not SR of PROMs |
| Content comparison of health related quality of life measures for cerebral palsy based on the international classification of functioning children and youth version (ICF-CY) | Without measurement properties |
| What outcome measures are used with children and youth with cerebral palsy? a systematic review of the literat ure | Unable to extract pediatric PROM data |
| Evaluating cough assessment tools: a systematic review | Unable to extract pediatric PROM data |
| Predictive Validity of Developmental Screening Questionnaires for Identifying Children with Later Cognitive or Educational Difficulties: A Systematic Review | Unable to extract pediatric PROM data |
| Evaluating the effectiveness and validity of scoring tools in healthcare | Unable to extract pediatric PROM data |
| The State of Patient-Reported Outcome Measures for Pediatric Patients with Inherited Retinal Disease | Unable to extract pediatric PROM data |
| Identifying the most suitable quality of life assessment measures to assess humanistic burden of acute otitis media in children | Unable to extract pediatric PROM data |
| The assessment of juvenile psychopathy: Strengths and weaknesses of currently used questionnaire measures | Unable to extract pediatric PROM data |
| Physical activity parenting: a systematic review of questionnaires and their associations with child activity levels | Without measurement properties |
| THE INFANT GASTROESOPHAGEAL REFLEX DISEASE QUESTIONNAIRE (I-GERQ-R): A META-ANALYSIS | Unable to extract pediatric PROM data |
| Health-related quality of life measurement in children and adolescents: a systematic review of generic and disease-specific instruments | Without recommendations |
| A review of cultural adaptations of screening tools for autism spectrum disorders | Without measurement properties |
| A review of the use of self-report assessment with young children | Unable to extract pediatric PROM data |
| Age-Appropriate Pediatric Sports Patient-Reported Outcome Measures and Their Psychometric Properties: A Systematic Review | Without measurement properties |
| GETTING SET UP FOR MEANINGFUL MEASUREMENT-BASED CARE: OUTCOMES, INSTRUMENT SELECTION, AND IMPLEMENTATION | Unable to extract pediatric PROM data |
| Patient-reported outcome measures (PROMs) in paediatric ophthalmology: a systematic review | Without measurement properties |
| Health-Related Quality-of-Life Instruments for Pediatric Patients with Diverse Facial Deformities: A Systematic Literature Review | Without measurement properties |
| Pulmonary symptoms in adolescent idiopathic scoliosis: a systematic review to identify patient-reported and clinical measurement instruments | Without measurement properties |
| A systematic review of health-related quality of life assessment for children and adolescents with chronic kidney disease | Unable to extract pediatric PROM data |
| Family‐based programmes for preventing smoking by children and adolescents | Without measurement properties |
| Knee Arthroscopy Cohort Southern Denmark (KACS): Protocol for a prospective cohort study | Without measurement properties |
| Trajectory of self-reported pain and function and knee extensor muscle strength in young patients undergoing arthroscopic surgery for meniscal tears: A systematic review and meta-analysis | Without measurement properties |
| Measurement properties of questionnaires assessing complementary and alternative medicine use in pediatrics: a systematic review | Unable to extract pediatric PROM data |
| Instruments to assess self-care among healthy children: A systematic review of measurement properties | Without recommendations |
| Criterion Validity and Applicability of Motor Screening Instruments in Children Aged 5-6 Years: A Systematic Review | Unable to extract pediatric PROM data |
| The psychometric properties of the Ages & Stages Questionnaires for ages 2-2.5: a systematic review | Unable to extract pediatric PROM data |
| Children's self-report of pain intensity: what we know, where we are headed | Without measurement properties |
| A systematic review of pediatric self-report symptom measures: Congruence with the theory of unpleasant symptoms | Without measurement properties |
| Asthma control questionnaires in the management of asthma in children: A review | Without measurement properties |
| Reliability and Validity of Physical Activity Instruments Used in Children and Youth with Physical Disabilities: A Systematic Review | Unable to extract pediatric PROM data |
| Content Validity of Patient-Reported Outcome Instruments used with Pediatric Patients with Facial Differences: A Systematic Review | Without recommendations |
| Medical care experiences of children with autism and their parents: A scoping review | Without measurement properties |
| Non‐pharmaceutical management of respiratory morbidity in children with severe global developmental delay | Without measurement properties |
| [A systematic review of evaluation studies on physical activity questionnaires for children and adolescents] | Unable to extract pediatric PROM data |
| Definitions of Pediatric Functional Abdominal Pain Disorders and Outcome Measures: A Systematic Review | Without measurement properties |
| Quality of life domains affected in children with developmental coordination disorder: a systematic review | Without measurement properties |

**eTable 3 Characteristics of the recommended PROMs**

| Study ID | Condition | Number of recommended proms | Names of the proms | Year developed | Country | Language versions | Time to complete (min) | Content of interest | Population | Mode of reporting | Number of items | Domains assessed |
| --- | --- | --- | --- | --- | --- | --- | --- | --- | --- | --- | --- | --- |
| Kusi-Mensah 2022 | Brain pathology | 5 | Behavior Rating Inventory of Executive Function, BRIEF(parent) | 2000 | / | 1.Portuguese 2.Luganda 3.Turkish 4.Spanish 5.Persian 6.Luganda 7.Afrikaans 8.Xhosa 9.Shona 10.Zulu 11.Chichewa 12.Sesotho 13.Setswana | / | Executive functions | 1.School-age children 2.Children 3.Adolescents | Parent report | / | 1.Inhibition 2.Flexibility 3.Emotional control (behavioural regulation index) 4.Initiative 5.Working memory 6.Planning/organization 7.Material organization 8.Monitoring |
|  |  |  | BRIEF(teacher) | 2000 | / | 1.Portuguese 2.Persian | / | Executive functions | 1.Children and adolescents 2.School-age children | Proxy report | / | 1.Inhibition 2.Flexibility 3.Emotional control (behavioral regulation index) 4.Initiative 5.Working memory 6.Planning/organization 7.Material organization 8.Monitoring |
|  |  |  | BRIEF(self) | 2000 | / | Portuguese | / | Executive functions | Adolescents | Self report | / | 1.Inhibition 2.Flexibility 3.Emotional control (behavioral regulation index) 4.Initiative 5.Working memory 6.Planning/organization 7.Material organization 8.Monitoring |
|  |  |  | BRIEF(preschool) | 2004 | / | 1.Spanish 2.Kannada | / | Executive functions | Pre-school children | Parent report | / | 1.Inhibition 2.Change 3.Emotional control 4.Working memory 5.Planning / organization |
|  |  |  | Vineland Adaptive Behavior Scales, VABS | 1992 | Indonesia | 1.Indonesian 2.Kannada 3.Vietnamese | / | Social functions | 1.Children 2.Pre-school children | Parent report | / | 1.Communication 2.Daily living skills 3.Socialization 4.Maladaptive behavior |
| Charles 2022 | Early-onset scoliosis | 1 | Early-Onset Scoliosis Questionnaire, EOSQ | 2011 | / | 1.English 2.Dutch 3.Turkish 4.Chinese 5.Persian 6.Arabic 7.Norwegian 8.German 9.Spanish | / | Quality of life | 0–10 years | Parent/proxy report | 24 | / |
| Marshall 2022 | Sport-Related Injuries | 6 | Youth Throwing Scale | / | / | / | / | Body functions | 10–18 years | Parent report | 18 | Physiological domain |
|  |  |  | Knee Injury and Osteoarthritis Outcome Score for Children, KOOS-Child | / | / | / | / | Body functions | 10–18 years | Parent report | 46 | 1.Physiological domain 2.Social domain 3.Physical domain |
|  |  |  | Pediatric International Knee Documentation Committee Subjective Knee Evaluation Form, Pedi-IKDC | / | / | / | / | Body functions | 10–18 years | Parent report | 10 | 1.Physiological domain 2.Physical domain |
|  |  |  | Pediatric Quality of Life Inventory, pedsql | / | / | / | / | Quality of life | 2–18 years | Parent report | 23 | 1.Physiological domain 2.Social domain 3.Physical domain 4.Psychological domain |
|  |  |  | Activities Scale for Kids, ASK | / | / | / | / | Body functions | 5–15 years | Parent report | 30 | Physical domain |
|  |  |  | Oxford Ankle Foot Questionnaire for Children, oxafq-C | / | / | / | / | Body functions | 5–16 years | Parent report | 14 | 1.Physiological 2.Social 3.Physical 4.Psychological |
| Mcgee 2022 | Endocrinology | 2 | Diabetes Quality of Life for Youth Scale | 1998 | 18 countries in Europe, Japan and North USA | 14 languages (not specified) | / | Quality of life | 10–18 years | Parent report | 21 | 1.Impact of diabetes 2.Worries about diabetes  3.Satisfaction with treatment 4.Satisfaction with life 5.Health perception |
|  |  |  | Problem Areas In Diabetes Scale - Child Version | 2014 | USA | English | / | Quality of life | 8–12 years | Self/parent report | 27 | 1.Emotional burden 2.Regimen specific distress 3.Negative emotions 4.Keeping up with chronic demands 5.Personal regimen-specific distress 6.Child regimen-specific distress |
| Schokman 2022 | Narcolepsy | 2 | Epworth Sleepiness Scale—Children and adolescent, ESS-CHAD | / | USA | English | / | Sleep functions | 1.Children 2.Adolescents | Parent report | / | / |
|  |  |  | Narcolepsy Severity Scale-Paediatric, NSS-P | / | France | French | / | Sleep functions | Children | Parent report | / | / |
| Smith 2022 | Neurodevelopmental disorders | 2 | Cerebral Palsy Quality of Life tool teenager slef-report, CP QOL teenager slef-report | / | / | 1.English 2.Turkish 3.Persian 4.Finnish 5.Polish 6.German | / | Quality of life | Teenager | Self report | / | / |
|  |  |  | Cerebral Palsy Quality of Life tool teenager primary caregiver,  CP QOL teenager primary care giver | / | / | 1.English 2.Turkish 3.Persian 4.Finnish 5.Polish 6.German | / | Quality of life | Teenager | Proxy report | / | / |
| Soler 2022 | Neurodevelopmental disorders | 1 | Participation and Sensory Environment Questionnaire- Home , PSEQ- H | / | / | / | / | Body functions | 1.Children  2.Adolescents | Proxy report | / | / |
| Mazefsky 2021 | Emotion Regulation and Reactivity | 7 | Children’s Emotional Adjustment Scale, CEAS | 2015 | / | / | / | Emotional functions | 1.Preschool child 2.Early Adolescent | Proxy report | 1.47 (child/adolescent) 2.29 (preschool) | 1.Temper Control 2.Anxiety Control 3.Social Assertiveness 4.Mood Repair |
|  |  |  | Emotion Dysregulation Inventory, EDI | 2018 | / | / | / | Emotional functions | 1.Toddler/Preschool  2.Child 3.Adolescent 4.Adult | Self/proxy report | 1.24(child/adolescent) 2.6(preschool) | 1.Reactivity 2.Dysphoria |
|  |  |  | Affective Reactivity Index, ARI | 2012 | / | / | / | Emotional functions | 1.Child 2.Adolescent 3.Adult | Self/proxy report | 7 | Irritability and a separate impairment item |
|  |  |  | Children’s Inventory of Anger, chia | 2000 | / | / | / | Emotional functions | 1.Child 2.Adolescent | Self report | 39 | 1.Total, Frustration 2.Physical Aggression 3.Peer Relationships 4.Authority Relations |
|  |  |  | Emotional Cultivation Scale, ECS | 2019 | / | / | / | Emotional functions | 1.Child 2.Adolescent | Self report | 9 | 1.Cultivating Emotion Strategies 2.Understanding Emotion Connotations |
|  |  |  | Aberrant Behavior Checklist- 2, ABC-2; | 2017 | / | / | / | Behavior | 1.Child 2.Adolescent 3.Adult | Proxy report | 58 | 1.Irritability 2.Social Withdrawal 3.Stereotypic Behavior 4.Hyperactive/ Noncompliance 5.Inappropriate Speech |
|  |  |  | Multidimensional Assessment of Preschool Disruptive Behavior, MAP-DB | 2014 | / | / | / | Behavior | 1.Infant 2.Toddler 3.Preschool age | Proxy report | 1.22 2.111(original preschool version) | 1.Temper loss  2.Noncompliance 3.Aggression 4.Low concern for others |
| Młyńczyk 2021 | Juvenile idiopathic arthritis | 4 | Euroqol Five Dimension Youth questionnaire, EQ-5D-Y | 2009 | / | English and over 50 different language versions | / | Quality of life | 8–15 years for self-report  4-15 years for parent proxy report | Self/parent/proxy report | 5 | 1.Mobility 2.Self-care 3.Usual activities 4.Pain/discomfort 5.Anxiety/depression |
|  |  |  | Pediatric quality of life inventory 3.0 rheumatology module, pedsql 3.0 rheumatology module | 2002 | / | English and different language versions | / | Quality of life | 5–18 years for self-report  2-18 years for parent proxy report | Self/parent/proxy report | 22 | 1.Pain  2.Hurt 3.Daily activities 4.Treatment 5.Worry 6.Communication |
|  |  |  | Pediatric quality of life inventory 4.0 Generic core, pedsql 4.0 Generic core scales | / | / | English and different language versions | / | Quality of life | 5–18 years for self-report  2–18 years for parent/ proxy report | Self/parent/proxy report | 23 | 1.Physical functioning 2.Emotional functioning 3.Social functioning 4.School functioning |
|  |  |  | Pediatric quality of life inventory 4.0 SF15 Generic core, pedsql 4.0 SF15 Generic core scales | / | / | English and different language versions | / | Quality of life | 5–18 years for self-report  2-18 years for parent proxy report | Self/parent/proxy report | 15 | 1.Physical functioning 2.Emotional functioning 3.Social functioning 4.School functioning |
| Andrei 2020 | Excessive daytime sleepiness | 1 | Pediatric Daytime Sleepiness Scale, PDSS | / | / | / | / | Sleep functions | 1.Children 2.Adolescents | Self report | / | / |
| GABRIELA 2021 | Upper-limb function | 3 | Performance of Upper Limb, PUL | 2013 | Italy | 1.Italian 2.English 3.Turkish | 15 min | Body functions | 1.Children 2.Adolescents | Proxy report | 22 | 1.Shoulder level 2.Elbow level  3.Distal level |
|  |  |  | Duchenne muscular dystrophy (DMD) Upper Limb patient-reported outcome measures (proms), DMD Upper Limb PROM | 2017 | Italy | 1.English 2.French 3.Dutch  4.German  5.Italian  6.Portuguese  7.Spanish  8.Turkish  9.Danish  10.Swedish | 10 min | Body functions | 1.Children 2.Adolescents | Self report | 32 | 1.Food 2.Self-care 3.Household and environment 4.Leisure and communication |
|  |  |  | Revised Upper Limb Module, RULM | 2017 | USA | 1.English 2.Italian | 20min | Body functions | 1.Children 2.Adolescents | Proxy report | 20 | / |
| Mahakwe 2021 | Cancer | 10 | 100-mm Visual Analogue scale, VAS | / | / | / | / | Emotional functions | 7–18 years | Self report |  | / |
|  |  |  | Hospital Anxiety and Depression Scale—Anxiety, HADS-A | / | / | / | / | Emotional functions | 13–19 years | Self report | 14 | / |
|  |  |  | Kessler Psychological Distress Scale , K10 | / | / | / | / | Emotional functions | 15–25 years | Self report | 10 | / |
|  |  |  | Patient-Reported Outcomes Measurement Information System , PROMIS | / | / | 1.English 2.Chinese | / | Quality of life | 7–18 years | / | / | / |
|  |  |  | Pedsql^TM^ 3.0 Brain Tumor module | / | / | / | / | Quality of life | 5–18 years for self-report 2–4 years for proxy-report | Self/proxy report | 24 | / |
|  |  |  | Pedsql^TM^ 3.0 Cancer Module | / | / | / | / | Quality of life | 2–18 years | Self/proxy report | 27 | / |
|  |  |  | Revised Child Manifest Anxiety Scale, RCMAS | / | / | / | / | Emotional functions | 6–17 years | Self report | 37 | / |
|  |  |  | Revised Child Manifest Anxiety Scale-2, RCMAS-2 | / | / | / | / | Emotional functions | 6–19 years | Self report | 40 | / |
|  |  |  | State Trait Anxiety Inventory (STAI) Trait and State Scale | / | / | / | / | Emotional functions | 12–20 years | Self report | 40 | / |
|  |  |  | State Trait Anxiety Inventory for Children, STAIC | / | / | / | / | Emotional functions | 8–18 years | Self report | 20 | / |
| Hajra 2020 | Negative Self‑Referential Emotions | 2 | Child-Adolescent Perfectionism Scale, CAPS | 2016 | 1.Canada 2.USA | / | / | Emotional functions | 1.Adolescents 2.Children | Self report | 22 | / |
|  |  |  | Children’s automatic Thoughts Scale, CATS | 2002 | Australia | / | / | Emotional functions | 1.Adolescents 2.Children | Self report | 40 | / |
| Holly 2020 | Epilepsy | 2 | Quality of Life in Childhood  Epilepsy, qolce-55 | 2015 | Canada | / | / | Quality of life | 4–18 years | Parent report | 55 | 1.Cognitive domain 2.Emotional domain 3.Social domain 4.Physical domain |
|  |  |  | Health-Related Quality of Life Measure for Children with Epilepsy ,cheqol | 2003 | Canada | / | / | Quality of life | 6–15 years | Self/parent report | 25 | 1.Interpersonal/social domain  2.Intrapersonal/emotional domain  3.Present worries/Concerns  4.Secrecy  5.Quest for normality (child only) or future worries and concerns (parent only) |
| Marson 2020 | Fractures | 4 | Patient- Reported Outcomes Measurement Information System Mobility, PROMIS Mobility | 2012 | USA | / | / | Body functions | 8–17 years | Self/proxy report | / | / |
|  |  |  | Patient- Reported Outcomes Measurement Information System Upper Extremity, PROMIS Upper Extremity | 2012 | USA | / | / | Body functions | 8–17 years | Self/proxy report | / | / |
|  |  |  | Activity Scale for Kids, ASK | 1995 | Canada | / | / | Body functions | 5–15 years | Self/proxy report | 30 | / |
|  |  |  | Euroqol Five Dimension Youth questionnaire, EQ- 5D- Y | / | The Netherlands | / | / | Quality of life | 8–18 years | Self report | 6 | / |
| Samantha 2019 | Solid organ transplantation | 1 | Pediatric Quality of Life Inventory Generic Core Scales, pedsql™ Generic Core Scales | / | USA | / | / | Quality of life | 8–18 years | / | 23 | 1.Physical functioning 2.Emotional functioning 3.Ssocial functioning 4.School functioning |
| Kathryn 2019 | Pain | 3 | 11-point numeric rating scale ,/S-11 | / | / | 1.English 2.Catalan 3.French 4.German 5.Spanish. | / | Sensory functions | 3–20 years | Self report | / | / |
|  |  |  | Faces Pain Scale–Revised, FPS-R | / | / | 1.English 2.Catalan 3.Portuguese 4.Spanish 5.Thai 6.German 7.French | / | Sensory functions | 3–19 years | Self report | / | / |
|  |  |  | Color Analogue Scale, CAS | / | / | 1.English 2.Thai 3.Spanish 4.Catalan 5.French | / | Sensory functions | 5–16 years | Self report | / | / |
| Natalie 2019 | Mental Health | 5 | Y-QOL-R | 2002 | USA | / | / | Quality of life | Adolescents | Self report | 49 | 1.Self relationships 2.Environment 3.General quality of life |
|  |  |  | Y-QOL.30.1 | 2005 | USA | / | / | Quality of life | Adolescents | Self/parent report | 30 | 1.Somatic, social isolation, aggression, conduct problems 2.Depression/anxiety 3.Hyperactivity/Distractibility |
|  |  |  | GHQ-12 | 2003 | Australia | / | / | Emotional functions | 11–15 years | Self report | 12 | 1.Psychological distress and well-being |
|  |  |  | ORS | 2006 | USA | / | / | Emotional functions | 13–17 years | Self/parent report | 5 | 1.Individual domain 2.Social domain 3.Relational domain |
|  |  |  | YP CORE | 2016 | UK | / | / | Body functions | 13–16 years | Self report | 10 | 1.Wellbeing symptoms/functioning 2.Risk (to self) |
| Yang 2019 | Oral Health | 3 | Scale of Oral Health Outcomes for 5-year-old children, SOHO-5 | 2012 | UK | / | / | Quality of life | Schoolchildren | Self report | 7 | / |
|  |  |  | Pediatric Oral Health–Related Quality of Life–Parent Report on Child, POQL-P | 2011 | USA | / | / | Quality of life | Schoolchildren | Parent report | 10 | / |
|  |  |  | Michigan Oral Health–Related Quality of Life Scales–Parent/Guardian version, mohrqol-PG | 2003 | USA | / | / | Quality of life | Children with or without early childhood caries | Parent/proxy report | 9 | / |
| Mathews 2019 | Maternal/Caregiver Attachment | 6 | Maternal Attachment Inventory , MAI | 1994 | USA | English | / | Emotional functions | / | Self report | 26 | / |
|  |  |  | Postpartum Bonding Questionnaire , PBQ | 2006 | England | English | / | Emotional functions | / | Self report | 25 | / |
|  |  |  | Maternal Postpartum Attachment Scale, MPAS | 1998 | Australia | English | 15-20 minutes | Emotional functions | / | Self report | 19 | / |
|  |  |  | Paternal Postnatal Attachment Scale, PPAS | 2008 | Australia | English | 15-20 minutes | Emotional functions | / | Self report | 19 | / |
|  |  |  | Maternal Feelings Questionnaire, Troy | 1993 | USA | English | / | Emotional functions | / | Self report | 3 | / |
|  |  |  | Mother-to-Infant Bonding Questionnaire, MIBS | 2005 | / | English | 2-3 minutes | Emotional functions | / | Self report | 8 | / |
| Zaror 2019 | Oral Health | 2 | Early Childhood Oral Health Impact Scale, ECOHIS | / | / | 1.English 2.Chinese 3.Spanish 4.Portuguese(Portugal,Luanda) 5.French 6.Persian 7.Arabic 8.Turkish 9.Lithuanian 10.Kiswahili 11.Kannada 12.Malayalam 13.Malay | / | Body functions | 0–5 years | Proxy report | 13 | 1.Symptoms  2.Function 3.Psychology 4.Social 5.Parental distress 6.Family function |
|  |  |  | Child Perceptions Questionnaire 11–14 | / | / | 1.English 2.Spanish 3.Portuguese 4.German 5.Arabic 6.Malay 7.Thai 8.Italian 9.Cambodian 10.Danish 11.Korean 12.Telugu | / | Body functions | 11–14 years | Self report | 37 | 1.Oral symptoms 2.Functional limitations 3.Emotional well-being 4.Social well-being |
| Stahlschmidt 2019 | Pain | 2 | Child Self-Efficacy Scale, CSES | 1986 | / | English | / | Sensory functions | 8–18 | Self report | 7 | / |
|  |  |  | Pain Beliefs Questionnaire , PBQ | 1984 | / | 1.English 2.Dutch | / | Sensory functions | 7–18 | Self report | 12 | / |
| Richard 2018 | Otitis media with effusion | 3 | Evaluation of Children’s Listening and Processing Skills ,eclips | / | / | / | / | Body functions | Children (6-11y) | Parent report | 38 | 1.Speech & Auditory Processing,  2.Environmental & Auditory Sensitivity,  3.Language/Literature/ Laterality 4.Pragmatic & Social Skills 5.Memory & Attention. |
|  |  |  | Auditory Questionnaire, littlears | / | / | / | / | Behavior | Young children(0-2y) | Parent/proxy report | 35 | 1.Auditory responsiveness to stimuli like music 2.Speech, environmental sounds 3.Capacity to connec |
|  |  |  | Evaluation of Aural/Oral Performance of children, PEACH | / | / | / | / | Body functions | Preschool | Parent report | 13 | 1.Ability to hear and communicate (in quiet, in different noisy situations, and when using a telephone) 2.Responsiveness to different sound |
| Sarri 2018 | Sickle cell disease | 2 | Pediatric Quality of Life sickle cell disease, pedsql™ SCD | / | / | / | / | Quality of life | 1.Children 2.Adolescents | Self report | 43 | / |
|  |  |  | Sickle Cell Disease Pain Burden Interview-Youth , SCPBI-Y | / | / | / | / | Sensory functions | 1.Children 2.Adolescents | Self report | 7 | / |
| Yolanda 2017 | Non-suicidal self-injury | 2 | Alexian Brothers Urge to Self-Injure Scale; FASM, Functional Assessment of Self -Mutilation,ABUSI | 2010 | USA | / | / | Body functions | Adolescents | Self report | / | Unidimensional |
|  |  |  | Impulse, Self-harm and Suicide Ideation Questionnaire for Adolescents,ISSIQ-A | 2015 | Portugal | / | / | Behavior | Adolescents | Self report | / | 1.Impulsivity 2.self-injury 3.risk behaviors 4.suicidal ideation |
| LIMPERG 2017 | Haemophilia | 1 | Canadian Haemophilia Outcomes-Kids Life Assessment Tool, CHO-KLAT | 2004 | Canada | English | / | Quality of life | Children 4–18 years | Self report | 35 | / |
| Dietvorst 2017 | Knee ligament injury | 1 | Pediatric International Knee  Documentation Committee, Pedi-IKDC | / | / | / | / | Body functions | Children with knee ligament injuries | / | 18 | 1.Symptoms 2.functioning 3.Sport activities |
| Lucendo 2017 | Eosinophilic oesophagitis | 1 | Pedsql eosinophilic esophagitis module, Peds-qol eoe Module | 2013 | / | / | / | Quality of life | Children | Self/parent/proxy report | 33 | 1.Symptoms I 2.Symptoms II  3.Treatment 4.Worry 5.Communication 6.Food and eating 7.Food feelings |
| Nobuaki 2016 | Cerebral palsy | 3 | ABILOCO-Kids | / | / | / | 5min | Body functions | 1.Children 2.Adolescents | Proxy report | 10 | / |
|  |  |  | Gillette Functional Assessment Questionnaire | / | / | / | 5min | Body functions | 1.Children 2.Adolescents | Self/proxy report | / | / |
|  |  |  | Functional Mobility Scale | / | / | / | 5min | Body functions | 1.Children 2.Adolescents | Self/proxy report | / | / |
| Ji 2016 | Sleep disturbances | 2 | Cleveland Adolescent Sleepiness Questionnaire, CASQ | 2007 | / | / | / | Sleep functions | Adolescents(10-17y) | Self/parent report | / | / |
|  |  |  | Chronic Sleep Reduction Questionnaire, CSRQ | 2008 | / | 1.Dutch 2.English | / | Sleep functions | Adolescents | Self/parent report | / | / |
| HUSSEIN 2015 | Asthma | 1 | Paediatric Asthma Quality of Life Questionnaire, PAQLQ | / | / | 1.English  2.Spanish  3.Dutch  4.French  5.Portuguese  6.Mandarin  7.Malay  8.Filipino | 10min | Quality of life | 7-17y | Self/parent/proxy report | 23 | 1.Activity limitation,  2.Symptoms  3.Emotional function |
| Jennifer 2015 | Autism | 2 | Child Behavior Checklist, CBCL | / | / | / | / | Behavior | Children (1.5-18y) | Proxy report | 118 | / |
|  |  |  | Home Situations Questionnaire—Pervasive Developmental Disorders version, HSQ-PDD | / | / | / | / | Behavior | Children (3-14y) | Proxy report | 25 | / |
| Myer 2015 | Dysphagia | 4 | Dysphagia in Multiple Sclerosis, DYMUS | 2009 | / | / | / | Quality of Life | Self report >12 | Self report | 10 | / |
|  |  |  | Dysphagia Symptom Questionnaire, DSQ | 2013 | / | / | / | Body functions | >12 | Self report | 3 | / |
|  |  |  | Symptom Questionnaire for eosinophilic esophagitis | 2008 | / | / | / | Body functions | 1.Parent report: 2-7 2.Self report: 8-17 | Self/parent report | 6 | / |
|  |  |  | Pediatric Quality of Life Inventory Gastrointestinal Symptoms Module, pedsql GI Module | 2012 | / | / | / | Quality of life | 1.Parent report: 2-18 2.Self report: 5-18 | Self/parent report | 74 | / |
| SANCHEZ 2015 | Feeding disorders | 1 | Behavioral Pediatrics Feeding Assessment Scale, BPFAS | / | / | English | / | Body functions | 7 months-13 years old | Proxy report | 35 | 1.Oral motor or dysphagia 2.Selectivity by type 3.Selectivity by texture 4.Food refusal |
| Alison 2014 | Fatigue | 5 | Pediatric Quality of Life Inventory  Multidimensional Fatigue Scale,pedsql MFS-child | / | / | / | 5min | Quality of life | 5-18y | Self report | 36 | 1.General fatigue 2.Sleep/rest fatigue 3.Cognitive fatigue 4.otal fatigue |
|  |  |  | Pediatric Quality of Life Inventory  Multidimensional Fatigue Scale,pedsql MFS-parent | / | / | / | Not specified | Quality of life | 2-18y | Parent report | 4 | General fatigue |
|  |  |  | Fatigue Scale-Child,FS-C | / | / | / | 3–10 min | Exercise tolerance functions | 7–12y | Self report | 38/42 | 1.Intensity 2.Frequency 3.Total fatigue |
|  |  |  | Fatigue Scale-Child,FS-A | / | / | / | 1.2–4 min 2.4–5 min | Exercise tolerance functions | 13-18y | Self report | 14 | Intensity |
|  |  |  | Fatigue Scale-Child, FS-P | / | / | / | Not specified | Exercise tolerance functions | 7-18y | Self report | 32/35/36 | 1.Intensity 2.Total |
| Griffiths 2014 | Burn | 3 | Perceived Stigmatisation Questionnaire, PSQ | / | USA | English | / | Behavior | Paediatric and adolescent burn patients (aged 8–18 y) | Self report | 21 | / |
|  |  |  | Social Comfort Questionnaire, SCQ | / | USA | English | / | Social functions | Paediatric and adolescent burn patients (aged 8–18 y) | Self report | 8 | / |
|  |  |  | Children Burn Outcomes Questionnaire, CBOQ | / | USA | English | / | Body functions | Paediatric and adolescent burn patients (aged 11–18 y) | Self report | 52 | / |
| Rainey 2014 | Disability(participation) | 1 | Child and Adolescent Scale of Participation, CASP | 2004 | / | 1.English 2.Chinese | 10–15 min | Social functions | Patients > 5 years with brain injury | Self/parent report | 20 | 1.Home 2.School 3.community 4.home and community living activities |
| Zhang 2014 | Chronic disease | 1 | Transition Readiness Assessment Questionnaire, TRAQ | / | / | English | / | Quality of life | Adolescent | Self report | 33 | / |
| Chien 2013 | Disability(hand use) | 3 | Participation and Enjoyment/Preferences for Activities of Children, CAPE/PAC | / | / | / | 30–45min (CAPE) 15min (PAC) | Social functions | Children with/without disabilities | Self/parent/proxy report | 55 | 1.Recreational 2.Active physical, 3.Social 4.Skill-based 5.Self-improvement) |
|  |  |  | School Function Assessment-Participation section, SFA-P | / | / | / | 5-10min | Social functions | Children with disabilities | Proxy report | 6 | Elementary school life domains:  1.Classroom 2.Playground/recess 3.Transportation, 4.Bathroom/toileting 5.Transitions 6.Mealtime/snack time |
|  |  |  | Children Participation Questionnaire, CPQ | / | / | / | / | Social functions | Children with/without disabilities | Parent report | 44 | 1.Activities of daily living, 2.Instrumental activities of daily living 3.Play 4.Leisure 5.Social participation  6.Eeducation |
| Paalman 2013 | Externalizing Mental Health Problems | 3 | Behavioral Assessment for Children of African Heritage, BACAH | / | / | / | / | Emotional functions | / | Self/parent/proxy report | / | / |
|  |  |  | Strengths and Difficulties Questionnaire, SDQ | / | / | / | / | Emotional functions | / | Self/proxy report | 25 | / |
|  |  |  | Adjustment Scales for Children and Adolescents, ASCA | / | / | / | / | Emotional functions | / | Self report | / | / |
| Noyes 2011 | Health-Related Quality of Life and Resource Allocation | 4 | Euroqol Five Dimension Youth questionnaire, EQ-5D-Y | / | / | / | / | Quality of life | / | Self report | / | / |
|  |  |  | Euroqol Five Dimension questionnaire Child Dutch | / | / | / | / | Quality of life | / | Self/proxy report | / | / |
|  |  |  | EQ-5D version extended with cognitive dimension, EQ-5D+C | / | / | / | / | Quality of life | / | Self/proxy report | / | / |
|  |  |  | Euroqol Five Dimension questionnaire adult version, EQ-5D adult | / | / | / | / | Quality of life | / | Self report | / | / |
| CATHERINE 2010 | Cerebral palsy | 2 | Activities Scale for Kids – Performance version | / | / | / | / | Body functions | Children | / | / | / |
|  |  |  | Children’s Assessment of Participation and Enjoyment, CAPE⁄PAC | / | / | / | / | Body functions | 6-12 years | / | / | / |
| Stacey 2010 | Cerebral palsy | 2 | Caregiver priorities and child health index of life with disabilities, CPCHILD | / | / | / | 20-30min | Quality of life | 5-12 years | Parent/proxy report | / | 1.Personal care 2.Positioning,transferring & mobility 3.Comfort emotions and behaviour 4.Communication and social interaction 5.Health 6.Overall quality of life |
|  |  |  | Psychometric properties of the quality of life questionnaire for children with CP., CP QOL-Child | / | / | / | 15-25min | Quality of life | 4-12 years | Self/parent/proxy report | 119 | 1.Social well-being and acceptance 2.Participation and physical health 3.Functioning 4.Emotional well-being 5.Pain and impact of disability 6.Access to services 7.Family |
| Stinson 2006 | Pain | 2 | Faces Pain Scale-Revised, FPS-R | 2001 | / | / | / | Sensory functions | / | Self report | / | 1.Acute procedural pain 2.Post-operation pain 3.Disease related pain |
|  |  |  | Visual analogue scale | 1977 | / | / | / | Sensory functions | / | Self report | / | 1.Acute pain 2.Procedural pain 3.Disease-related pain 4.Recurrent/chronic pain |

**eTable 4 Results of measurement properties of recommended PROMs**

| **Name of PROMs** | **Content validity** | **Structural validity** | **Internal consistency** | **Cross-cultural validity\ Measurement invariance** | **Reliability** | **Measurement error** | **Criterion validity** | **Hypotheses testing for construct validity** | **Responsiveness** | **Results from other criteria except COSMIN** | **Measurement properties of the criteria** |
| --- | --- | --- | --- | --- | --- | --- | --- | --- | --- | --- | --- |
| Behavior Rating Inventory of Executive Function, BRIEF(parent) | RoB -3 o/a 1 adaptation study of ‘inadequate’ quality, and 1 instrument dev. study of ‘inadequate’ quality. Hence VERY LOW | (?)  Did not do EFA or CFA only PCA | (+) Cronb. Alpha 0.93 | (?)  No MGCFA performed, rather did PCA | (-) ICC ranged 0.5 to 0.57 | (?)  MIC not defined | / | (-) for convergent validity;  +  for discriminant validity for ADHD | / | / | / |
| BRIEF(teacher) | / | (+) CFI between 0.99 - 1.0, RMSEA bet. 0.0 - 0.33 | (+) Cronb. Alpha between 0.79 - 0.99 | / | / | / | / | (+) results in line with 1 hypothesis (1+ convergent validity for WISC coding) | / | / | / |
| BRIEF(self) | (+) based on best result rating rule. Thus RoB -3 o/a 1 adaptation study of ‘inadequate’ quality, and 1 instrument development study of ‘inadequate’ quality. Hence ‘VERY LOW’ qual. Of evidence | (?) Did not do CFA |  | / | / | / | / | / | / | / | / |
| BRIEF(preschool) | / | (?)  Did not report results of CFA or even EFA | (+) Cronb. Alpha between 0.60 and 0.93 | (?)  no MGCFA performed | (+) ICC for inter-rater bet. 0.90 to 0.94 | / | / | (+) results in line with both hypothesis | / | / | / |
| ﻿Vineland Adaptive Behavior Scales, VABS | (+) based on best result rating rule. Thus RoB -3 o/a 2 adaptation studies both of ‘inadequate’ quality, and 1 instrument development study of ‘inadequate’ quality. | (-) CFI 0.93 - 0.97, but RMSEA was 0.115 - 0.095 | (+) Cronb. Alpha between 0.76 to 0.99 | (?) No MGCFA performed | (+) ICC for test-retest bet 0.83 to 0.90, ICC for inter-rater bet. 0.93 to 0.97 | / | / | (+++) sufficient；all 3 discriminant hypothesis confirmed | / | / | / |
| Early-Onset Scoliosis Questionnaire, EOSQ | (+) | / | / | / | (+) | / | (+) | (?) | (?) | / | / |
| Youth Throwing Scale | floor and ceiling effects ,30% | / | Cronbach α = 0.93 | / | / | / | correlation between Youth Throwing Scale and existing validated evaluation methods | between healthy and injured players | minimal detectable change: 9.4 points | / | / |
| Knee Injury and Osteoarthritis Outcome Score for Children, KOOS-Child | confirmed through cognitive interviews | / | Cronbach α = 0.59–0.90 | / | / | / | / | 75% relevant items | standard error of measurement: 5.28–8.14 points;  smallest detectable change(group):1.73–2.66 | / | / |
| Pediatric International Knee Documentation Committee Subjective Knee Evaluation Form, Pedi-IKDC | preoperative scores used | / | Cronbach α = 0.91 | / | / | / | correlations with Child Health Questionnaire subscales, r = 0.20–0.6 | >75% relevant items | effect size = 1.39 improvement after knee surgery =29.1 points | / | / |
| Pediatric Quality of Life Inventory, PedsQL | / | / | Cronbach α = 0.86–0.91 | / | / | / | / | healthy children scored higher than children with chronic health conditions | standard error of measurement: 4.4 points | / | / |
| Activities Scale for Kids, ASK | / | / | / | / | / | / | correlation with clinician-reported ASK, r = 0.92; significant differences between mild, moderate, and severely disabled groups (P, 0.0001) for clinicians’ global ratings | correlation with chilchildhood health assessment questionnaire r = 0.81–0.82 | Predicted to worsen:  effect size =0.63–0.84 Predicted to improve:  effect size = 1.08–1.15 | / | / |
| Oxford Ankle Foot Questionnaire for Children, OxAFQ-C | / | / | / | / | / | / | / | / | / | / | / |
| Diabetes Quality of Life for Youth Scale | Doubtful | Very good | Very good | Very good | / | / | / | Very good | / | / | / |
| Problem Areas In Diabetes Scale - Child Version | Doubtful | Very good | Very good | Very good | / | / | / | Very good | / | / | / |
| Epworth Sleepiness Scale—Children and adolescent, ESS-CHAD | Inconclusive | / | Indeterminant | / | Sufficient | / | / | / | Indeterminant | / | / |
| Narcolepsy Severity Scale-Paediatric, NSS-P | Inconclusive | N/A | N/A | / | Indeterminant | / | / | / | Indeterminant | / | / |
| Cerebral Palsy Quality of Life tool teenager slef-report, CP QOL teenager slef-report | (+) | (?) | (?) | / | (-) | / | / | (+) | / | / | / |
| Cerebral Palsy Quality of Life tool teenager primary caregiver,  CP QOL teenager primary care giver | (+) | (?) | (?) | / | (?) | / | / | (+) | / | / | / |
| Participation and Sensory Environment Questionnaire- Home , PSEQ- H | (+) | (+) | (+) | / | (-) | (+) | (+) | (+) | / | / | / |
| Children’s Emotional Adjustment Scale, CEAS | Y | Y | Y | / | Y | / | / | / | / | Excellent | 1.Norms 2.Internal Consistency (Cronbach’s Alpha, Split Half) 3.Interrater Reliability 4.Test-Retest Reliability (Stability) 5.Repeatability 6.Content Validity 7.Construct Validity (e.g., Predictive, Concurrent, Convergent, and Discriminant Validity) 8.Discriminative Validity 9.Prescriptive Validity 10.Validity Generalization 11.Treatment Sensitivity 12.Clinical Utility |
| Emotion Dysregulation Inventory, EDI | Y | Y | Y | / | Y | / | / | / | / | Excellent | / |
| Affective Reactivity Index, ARI | Y | Y | Y | / | Y | / | / | / | / | Excellent | / |
| Children’s Inventory of Anger, ChIA | Y | Y | Y | / | Y | / | / | / | / | Excellent | / |
| Emotional Cultivation Scale, ECS | Y | Y | Y | / | Y | / | / | / | / | Excellent | / |
| Aberrant Behavior Checklist- 2, ABC-2; | Y | Y | Y | / | Y | / | / | / | / | Excellent | / |
| Multidimensional Assessment of Preschool Disruptive Behavior, MAP-DB | Y | Y | Y | / | Y | / | / | / | / | Excellent | / |
| EuroQol Five Dimension Youth questionnaire, EQ-5D-Y | / | / | / | / | (+) | / | / | / | / | / | / |
| Pediatric quality of life inventory 3.0 rheumatology module, PedsQL 3.0 rheumatology module | / | / | / | / | (+) | / | / | / | / | / | / |
| Pediatric quality of life inventory 4.0 Generic core, PedsQL 4.0 Generic core scales | / | / | / | / | (+) | / | / | / | / | / | / |
| Pediatric quality of life inventory 4.0 SF15 Generic core, PedsQL 4.0 SF15 Generic core scales | / | / | / | / | (+) | / | / | / | / | / | / |
| Pediatric Daytime Sleepiness Scale, PDSS | / | / | Y | / | / | / | / | / | / | Total 14/16/17/19/20 | 1.Quality 2.External validity 3.Internal validity  4.Power of the report |
| Performance of Upper Limb, PUL | Very good | (+) | (+) | / | (+) | (?) | (+) | (+) | / | / | / |
| Duchenne muscular dystrophy (DMD) Upper Limb patient-reported outcome measures (PROMs), DMD Upper Limb PROM | Doubtful | (+) | (+) | / | (+) | / | / | (+) | / | / | / |
| Revised Upper Limb Module, RULM | Doubtful | (+) | (+) | / | (+) | / | / | / | (+) | / | / |
| 100-mm Visual Analogue scale, VAS | / | / | / | / | / | / | / | / | / | / | / |
| Hospital Anxiety and Depression Scale—Anxiety, HADS-A | / | / | / | / | / | / | / | / | / | / | / |
| Kessler Psychological Distress Scale , K10 | / | / | / | / | / | / | / | / | / | / | / |
| Patient-Reported Outcomes Measurement Information System , PROMIS | / | / | / | / | / | / | / | / | / | / | / |
| PedsQLTM 3.0 Brain Tumor module | / | / | / | / | / | / | / | / | / | / | / |
| PedsQLTM 3.0 Cancer Module | / | / | / | / | / | / | / | / | / | / | / |
| Revised Child Manifest Anxiety Scale, RCMAS | / | / | / | / | / | / | / | / | / | / | / |
| Revised Child Manifest Anxiety Scale-2, RCMAS-2 | / | / | / | / | / | / | / | / | / | / | / |
| State Trait Anxiety Inventory (STAI) Trait and State Scale | / | / | / | / | / | / | / | / | / | / | / |
| State Trait Anxiety Inventory for Children, STAIC | / | / | / | / | / | / | / | / | / | / | / |
| Child-Adolescent Perfectionism Scale, CAPS | / | / | / | / | / | / | / | / | / | / | / |
| Children’s automatic Thoughts Scale, CATS | / | / | / | / | / | / | / | / | / | / | / |
| Quality of Life in Childhood  Epilepsy, QoLCE-55 | / | (+++) | (+++) | / | / | / | / | (+++) | / | / | / |
| Health-Related Quality of Life Measure for Children with Epilepsy ,CHEQoL | (++) | (++) | (+/−) | / | (-) | / | / | (++) | / | / | / |
| Patient- Reported Outcomes Measurement Information System Mobility, PROMIS Mobility | + | / | / | / | / | / | / | / | / | / | / |
| Patient- Reported Outcomes Measurement Information System Upper Extremity, PROMIS Upper Extremity | + | / | not applicable | / | not applicable | / | / | / | / | / | / |
| Activity Scale for Kids, ASK | + | / | not applicable | / | not applicable | / | / | / | / | / | / |
| EuroQol Five Dimension Youth questionnaire, EQ- 5D- Y | + | / | / | / | / | / | / | / | / | / | / |
| Pediatric Quality of Life Inventory Generic Core Scales, PedsQL™ Generic Core Scales | / | / | α = 0.71‐0.88 | / | / | / | / | Correlations with PedsQL End   Stage Renal Disease; patients   reported significantly lower   health‐related QOL compared   to healthy children; parent‐child agreement (ICC = 0.39‐0.56 | / | / | / |
| 11-point numeric rating scale ,/S-11 | / | / | / | / | (+) | / | (+) | (+) | (+) | / | / |
| Faces Pain Scale–Revised, FPS-R | (+) | / | / | (?) | (?) | / | (+) | (+) | (+) | / | / |
| Color Analogue Scale, CAS | (?) | / | / | (?) | (+) | / | (?) | (+) | (+) | / | / |
| Y-QOL-R | (+) | (+) | (+) | / | (-) | / | / | (+) | / | / | / |
| Y-QOL.30.1 | (+) | / | (-) | / | (+/-) | / | / | / | / | / | / |
| GHQ-12 | / | (+) | (+) | / | / | / | / | (+) | / | / | / |
| ORS | / | / | (+) | / | (-) | / | / | (+) | / | / | / |
| YP CORE | / | / | (+) | / | (+/-) | / | / | (+) | (+) | / | / |
| ﻿Scale of Oral Health Outcomes for 5-year-old children, ﻿SOHO-5 | Y | / | / | / | Y | / | / | / | / | total score 15.0 | 1.Conceptual and Measurement Model 2.Reliability 3.Validity 4.Interpretability 5.Translation 6.Burden |
| ﻿Pediatric Oral Health–Related Quality of Life–Parent Report on Child, ﻿POQL-P | Y | / | / | / | Y | / | / | / | / | total score 14.0 | / |
| ﻿﻿Michigan Oral Health–Related Quality of Life Scales–Parent/Guardian version, MOHRQoL-PG | Y | / | / | / | Y | / | / | / | / | total score 13.0 | / |
| Maternal Attachment Inventory , MAI | / | / | / | / | a = 0.76-0.93 Test-retest = 0.61-0.65 | / | / | / | / | / | / |
| Postpartum Bonding Questionnaire , PBQ | / | / | / | / | a = .63-.79 Test-retest = .77-.95 | / | / | / | / | / | / |
| Maternal Postpartum Attachment Scale, MPAS | / | / | / | / | a = .78-.79 Test-retest = .48-.86 | / | / | / | / | / | / |
| Paternal Postnatal Attachment Scale, PPAS | / | / | / | / | a = .62-.81 Test-retest = .65-.70 | / | / | / | / | / | / |
| Maternal Feelings Questionnaire, Troy | / | / | / | / | a = .95 Test-retest = .94 | / | / | / | / | / | / |
| Mother-to-Infant Bonding Questionnaire, MIBS | / | / | / | / | a = .71 Test-retest = .57-.61 | / | / | / | / | / | / |
| Early Childhood Oral Health Impact Scale, ECOHIS | Y | / | / | / | Y | / | / | / | / | B A A U B A U A | 1.Conceptual clarity  2.Respondent burden  3.Reliability  4.Validity  5.Normative data  6.Item bias  7.Ceiling/ floor effects  8.Administrative burden |
| Child Perceptions Questionnaire 11–14 | Y | / | / | / | Y | / | / | / | / | B A A U A U U B | / |
| Child Self-Efficacy Scale, CSES | Y | / | / | / | Y | / | / | / | / | B A A U A U U B | / |
| Pain Beliefs Questionnaire , PBQ | / | / | Strong  Cronbach’s α=0.95–0.97 | / | / | / | / | / | / | / | / |
| Evaluation of Children’s Listening and Processing Skills ,ECLiPS | Good | Good | Strong Cronbach’s α=0.89 | / | / | / | / | / | / | / | / |
| Auditory Questionnaire, LittlEARS | (-) | / | (+) | / | (+) | / | (?) | (+) | / | / | / |
| Evaluation of Aural/Oral Performance of children, PEACH | (+) | / | (+) | / | 0 | / | (-) | (+) | / | / | / |
| Pediatric Quality of Life sickle cell disease, PedsQL™ SCD | (+++) | / | (?) | / | / | / | / | (+++) | / | / | / |
| Sickle Cell Disease Pain Burden Interview-Youth , SCPBI-Y | (+/-) | / | / | / | / | (+) | / | (-/+) | (+/?) | / | / |
| Alexian Brothers Urge to Self-Injure Scale; FASM, Functional Assessment of Self -Mutilation,ABUSI | （+/III) | / | （+/III) | / | / | (?) | / | （+/III) | （+/III) | / | / |
| Impulse, Self-harm and Suicide Ideation Questionnaire for Adolescents,ISSIQ-A | / | / | / | / | / | / | (+) | (++) | / | / | / |
| Canadian Haemophilia Outcomes-Kids Life Assessment Tool, CHO-KLAT | / | / | / | / | / | / | (?) | (+) | / | / | / |
| Pediatric International Knee  Documentation Committee, Pedi-IKDC | / | / | / | / | / | / | (?) | / | (+) | / | / |
| PedsQL eosinophilic esophagitis module, Peds-QoL EoE Module | / | (+) | (+) | / | / | / | / | / | (?) | / | / |
| ABILOCO-Kids | / | (+) | (+/-) | (+) | / | / | / | / | / | / | / |
| Gillette Functional Assessment Questionnaire | Y | / | / | / | Y | / | / | / | / | Validity determined by  comparing between disease  Severity  Reliability determined by  ICC=0.84-0.95 | / |
| Functional Mobility Scale | / | (++) | (++) | / | / | / | (++) | / | / | / | / |
| Cleveland Adolescent Sleepiness Questionnaire, CASQ | / | (+++) | (+++) | / | / | / | / | (+++) | (+++) | / | / |
| Chronic Sleep Reduction Questionnaire, CSRQ | Item generation via literature review and expert opinion. Description of item selection/reduction, measured items, target population. | / | Cronbach’s alpha = 0.914 | / | / | / | / | Scores related to mumultiple sclerosis patients with reported swallowing dysfunction showed elevated DYMUS against MS patients with no dysfunction (P < .0001). | / | / | / |
| Paediatric Asthma Quality of Life Questionnaire, PAQLQ | / | / | / | / | / | / | / | / | / | / | / |
| Child Behavior Checklist, CBCL | / | / | / | / | / | / | / | / | / | / | / |
| Home Situations Questionnaire—Pervasive Developmental Disorders version, HSQ-PDD | Item generation via literature review, expert opinion and refinement via feedback, focus and cognitive interviews. Target population defined. |  | Cronbach’s alpha reported >0.70 for all scales, >0.90 for total score (exception: Medicine for Child Self Report) | / | / | / | / | Greater disease-specific  symptoms score correlate  with lower overall generic  health related quality of life (P < .001). Omnibus analysis of variance F tests for known-groups validity (P< 0.001). | / | / | / |
| Dysphagia in Multiple Sclerosis, DYMUS | Based on existing instruments | Factor analysis – eigenvalues >1; five-factor solution accounted for 55% of cumulative variance; Tucker and Lewis’ Reliability Coefficient=0.96; root mean square error of approximation=0.05 Clinical samples (feeding problems, autism spectrum disorders, type 1 diabetes, cystic fibrosis, eosinophilic gastrointestinal disorders)  score higher than normative samples Responsive to therapy change | Cronbach’s α 0.74–0.88 0.65–0.80 0.72–0.87 0.81–0.93 | / | Pearson’s r 0.82–0.85 0.88–0.91 | / | / | / | / | / | / |
| Dysphagia Symptom Questionnaire, DSQ | (+++) | (?) | (?) | (+) | (+++) | (?) | / | (+++) | (?) | / | / |
| Symptom Questionnaire for eosinophilic esophagitis | (+++) | (+) | (?) | (+) | (+++) | (?) | / | (+++) | (?) | / | / |
| Pediatric Quality of Life Inventory Gastrointestinal Symptoms Module, PedsQL GI Module | (+++) | (++) | (-) | (+) | (?) | (?) | / | (+) | (?) | / | / |
| Behavioral Pediatrics Feeding Assessment Scale, BPFAS | (+++) | (+) | (?) | (?) | (-) | (?) | / | (+) | (?) | / | / |
| Pediatric Quality of Life Inventory  Multidimensional Fatigue Scale,PedsQL MFS-child | (+++) | (++) | (?) | (+) | (?) | (?) | / | (+) | (?) | / | / |
| Pediatric Quality of Life Inventory  Multidimensional Fatigue Scale,PedsQL MFS-parent | / | / | / | / | / | / | / | / | / | / | / |
| Fatigue Scale-Child,FS-C | / | / | / | / | / | / | / | / | / | / | / |
| Fatigue Scale-Child,FS-A | / | / | / | / | / | / | / | / | / | / | / |
| Fatigue Scale-Child, FS-P | + | ± | ++ | / | ± | / | / | (++) | / | / | / |
| Perceived Stigmatisation Questionnaire , PSQ | (+) | Y | (+) | / | / | / | / | (+) | Y | / | 1.Content validity 2.Internal consistency 3.Construct validity 4.Reproducibility 5.Responsiveness 6.Floor & ceiling effects 7.Interpretability |
| Social Comfort Questionnaire , SCQ | Excellent | / | Poor to excellent | / | / | / | Excellent | Excellent | / | / | / |
| Children Burn Outcomes Questionnaire , CBOQ | Excellent | / | Excellent | / | / | / | Adequate | Excellent | Adequate | / | / |
| Child and Adolescent Scale of Participation, CASP | Excellent | / | Poor to excellent | / | / | / | Excellent | Excellent | / | / | / |
| Transition Readiness Assessment Questionnaire, TRAQ | +++ | ++ | ++ | / | / | / | / | / | / | / | / |
| Participation and Enjoyment/Preferences for Activities of Children, CAPE/PAC | +++ | +++ | +++ | +/- | / | / | ++ | ++ | / | / | / |
| School Function Assessment-Participation section, SFA-P | ++ | ? | ? | ? | / | / | / | + | / | / | / |
| Children Participation Questionnaire ,CPQ | / | / | / | / | Test–retest reliability: 69.8%–99.7% | / | / | Convergent validity compared to KIDSCREEN: r= -0.52–0.55 (different domains); Convergent validity compared to Cantril-ladder: r= -0.37–0.1 (different domains); Convergent validity compared to CFQ: r= -0.625 to -0.279 (different domains) | / | / | / |
| Behavioral Assessment for Children of African Heritage, BACAH | / | / | / | / | Test-retest reliability: IntInterclass correlation coefficients= -0.25–1.00 | / | / | Convergent validity compared to TNO-AZTNO-AZL (Netherlands Organisation for Applied Scientific Research Academic Medical Centre) Children’s Quality Of Life Questionnaire:  r = -0.54 to -0.15 for different domains Convergent validity compared to TNO-AZL (Netherlands Organisation for Applied Scientific Research Academic Medical Centre) Children’s Quality Of Life Questionnaire  r = -0.712 to -0.227 for different domains. Construct validity: mean correlation between contextually similar domains with TNO-AZTNO-AZL (Netherlands Organisation for Applied Scientific Research Academic Medical Centre) Children’s Quality Of Life Questionnaire =0.55 | / | / | / |
| Strengths and Difficulties Questionnaire, SDQ | / | / | / | / | / | / | / |  | / | / | / |
| Adjustment Scales for Children and Adolescents, ASCA | / | / | / | / | / | / | Criterion validity: EQ-5D (index score) compared to clinicians’ score r = 0.33; | Convergent validity: EQ-5D (index score) compared to Child HeChild Health Questionnaire-Parent Form (different domains): r = 0.30–0.64;  EQ-5D (index score) compared to Child HChild Health and Illness Profile - Child Edition (different domains):  r= -0.24–0.51.  Convergent validity: EQ-5D (index score) compared to the the Short Form-36 health survey questionnaire (different domains) r = 0.29–0.71; | / | / | / |
| EuroQol Five Dimension Youth questionnaire, EQ-5D-Y | 0.99 | / | / | / | / | / | / | / | / | / | / |
| EuroQol Five Dimension questionnaire Child Dutch | 0.30–0.62 | / | / | / | / | / | / | / | / | / | / |
| EQ-5D version extended with cognitive dimension, EQ-5D+C | Caregiver rated importance of items × = 3.95 on 6 point ordinal scale (SD 0.63, range 2.67-4.90) | / | α = 0.74-0.93 across all domains | / | / | / | / | Higher GMFCS scores correlated with higher  CPCHILD scores (worse outcome) Convergent (Spearman’s r): (w.PEDI)  Self Care 0.607  Mobility 0.619  Social Function 0.518 | / | / | / |
| EuroQol Five Dimension questionnaire adult version, EQ-5D adult | Domains determined in consultation with children with CP and their parents (28 families) | / | α = 0.74-0.92 (caregivers) (n = 205) α = 0.80-0.90 (child-self report) (n = 53) | / | / | / | / | Global QOL r = 0.18-0.62 Global Health r = 0.21-0.56 | / | / | / |
| Activities Scale for Kids – Performance version,ASKp | (+) | / | / | / | (+) | / | / | (+) | (+) | / | / |
| Children’s Assessment of Participation and Enjoyment, CAPE⁄PAC | / | / | / | / | (+) | / | / | (+++) | (++) | / | / |
| Caregiver priorities and child health index of life with disabilities, CPCHILD | / | / | / | / | / | / | / | / | / | / | / |
| Psychometric properties of the quality of life questionnaire for children with CP., CP QOL-Child | / | / | / | / | / | / | / | / | / | / | / |
| Faces Pain Scale-Revised, FPS-R | / | / | / | / | / | / | / | / | / | / | / |
| Visual analogue scale | / | / | / | / | / | / | / | / | / | / | / |
